# ggtreeExtra: Compact Visualization of Richly Annotated Phylogenetic Data

**DOI:** 10.1093/molbev/msab166

**Published:** 2021-06-07

**Authors:** Shuangbin Xu, Zehan Dai, Pingfan Guo, Xiaocong Fu, Shanshan Liu, Lang Zhou, Wenli Tang, Tingze Feng, Meijun Chen, Li Zhan, Tianzhi Wu, Erqiang Hu, Yong Jiang, Xiaochen Bo, Guangchuang Yu

**Affiliations:** 1 Department of Bioinformatics, School of Basic Medical Sciences, Southern Medical University, Guangzhou, China; 2 Guangdong Provincial Key Laboratory of Proteomics, School of Basic Medical Sciences, Southern Medical University, Guangzhou, China; 3 Department of Biotechnology, Beijing Institute of Radiation Medicine, Beijing, China; 4 Division of Laboratory Medicine, Microbiome Medicine Center, Zhujiang Hospital, Southern Medical University, Guangzhou, China

**Keywords:** phylogeny, data integration, data visualization, software

## Abstract

We present the *ggtreeExtra* package for visualizing heterogeneous data with a phylogenetic tree in a circular or rectangular layout (https://www.bioconductor.org/packages/ggtreeExtra). The package supports more data types and visualization methods than other tools. It supports using the grammar of graphics syntax to present data on a tree with richly annotated layers and allows evolutionary statistics inferred by commonly used software to be integrated and visualized with external data. *GgtreeExtra* is a universal tool for tree data visualization. It extends the applications of the phylogenetic tree in different disciplines by making more domain-specific data to be available to visualize and interpret in the evolutionary context.

## Introduction

Phylogenetic trees are widely used in several biological fields, including comparative genomics, epidemiology, and microbiome. Integrating and visualizing phylogenetic trees with multidimensional associated data sets help to identify patterns and generate new hypotheses. For example, a recent research constructed a phylogenetic tree of SARS-CoV-2 and integrated the state information of initial diagnosis of Australian SARS-CoV-2 genomes and country information of the origin of the GISAID genomes to investigate origins and transmission pathways of the COVID-19 strains in Australia (Rockett et al. 2020). The development of high-throughput experimental technologies has expanded the scales of phylogenetic trees and associated data sets. For instance, a microbiome study may collect hundreds of samples and reconstruct a phylogenetic tree representing the evolutionary relationships of a microbial community composed of thousands of species. Associated data sets, such as the species abundance in each sample and the number or status of target genes for each species, can be incorporated and visualized on a phylogenetic tree to reveal new insights into factors that influence microbial community dynamics (Morgan et al. 2013; Segata et al. 2013; Ainsworth et al. 2015). However, integrating and visualizing multidimensional data with phylogenetic trees is still not an easy task. Over the past decade, several packages and web tools have been developed to integrate external data into phylogenetic trees, such as *iTOL* (Letunic and Bork 2019), *Evolview* (Subramanian et al. 2019), *Microreact* (Argimón et al. 2016), *ETE3* (Huerta-Cepas et al. 2016), and *GraPhlAn* (Asnicar et al. 2015). But these tools are developed mainly for certain fields and are difficult to apply to other research domains. We previously proposed two general methods for mapping and visualizing associated data on phylogeny, which were implemented in *ggtree* (Yu et al. 2018). The *geom_facet* function provided in *ggtree* (Yu et al. 2017) employs a modular design to separate tree visualization, data integration, and graph alignment (Yu et al. 2018). It allows us to visualize multiple associated data sets in different panels and serves as a general tool since there is no prerequisite for the input data type (Yu et al. 2018; Yu 2020). With the increasing type and scale of biological data, it is a new challenge to visualize richly layered phylogenetic data in the circular layout, which can display more data in a given space. However, the *geom_facet* function does not work with a circular layout. To fully extend *ggtree* to support the visualization of multisource phylogenetic data in the era of big data, especially for circular layout, we developed the *ggtreeExtra* package. The *ggtreeExtra* package allows progressively representing taxon-specific features on external panels of a phylogenetic tree and helps users to explore and compare different heterogeneous data sets in the evolutionary context. The *ggtreeExtra* package has been released within the Bioconductor project (Gentleman et al. 2004) and it is available at https://bioconductor.org/packages/ggtreeExtra.

## Results

**Fig. 1.**
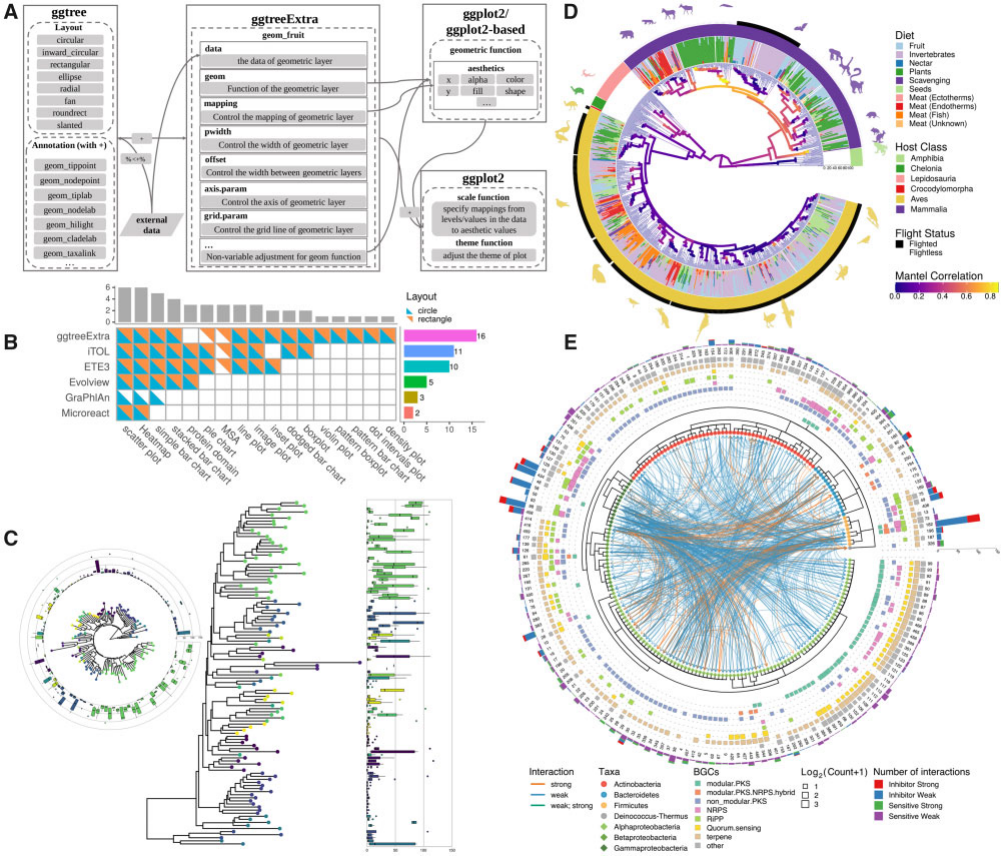
The design and features of the *ggtreeExtra* package. (*A*) The overall design of the *ggtreeExtra* package; (*B*) comparison of visualization methods for tree annotation (i.e., tree and data graphic alignment) supported by *ggtreeExtra* and other tools; (*C*) visualizing associated data (e.g., distribution of species abundance as boxplot) with a phylogenetic tree side by side or on the external ring (inset on the left); (*D*) using subplots and images as insets on a phylogenetic tree to present taxon-specific structural feature and summary statistics; (*E*) illustration of representing multidimensional data sets on an inward circular phylogenetic tree with chord diagram incorporated to display inter-relationships. The *ggtreeExtra* package supports both rectangular and circular layouts and allows transformation between different layouts (*C*). Multiple data sets can be integrated and a variable can be mapped to visual characteristics to visualize another type of data (CDE), such as using taxon information to color silhouette images (*D*).

The *ggtreeExtra* package implemented a layer function, *geom_fruit*, which is a universal function that aligns graphic layers to a phylogenetic tree (fig. 1*A*; supplementary table S1, Supplementary Material online). It can internally reorder associated data based on the structure of a phylogenetic tree, visualize the data using specific geometric layer function with user-provided aesthetic mapping and nonvariable setting, and the graphic layer will be displayed with the tree side by side (i.e., right-hand side for rectangular layout or external ring for circular layout; fig. 1*C*) with perfect alignment. Different data graph layers can be added to a tree progressively. For example, *geom_fruit* is able to display a heatmap and a bar plot to the outer rings of an annotated phylogenetic tree to compare microbial abundance across different body sites of humans (supplementary fig. S7, Supplementary Material online). These two layers were automatically aligned to the circular phylogenetic tree and were displayed on different external rings. The number of external rings is not strictly limited and the user is free to visualize several associated data sets using different geometric layers on different external rings. Each data set is visualized on an independent ring layer, and multiple ring layers are stacked on a circular phylogenetic tree, which makes the *ggtreeExtra* package particularly useful for layering different data sets to create highly informative tree graphics. For example, multiple heatmaps and bar chart layers were compactly displayed on the circular tree to represent the status of the gene, metabolic capacity, and genome size of 963 bacteria and archaea species (supplementary fig. S8, Supplementary Material online).

Unlike other tools, *ggtreeExtra* was developed based on the grammar of graphics (Wilkinson 2012) and allowed users to map variables of associated data to visual attributes of the outer ring graphic layer at a high level of abstraction (supplementary figs. S3, S4, and S7, Supplementary Material online). The geometric layers defined in *ggplot2* (Wickham 2016) and its extensions can be used in the *geom_fruit* function. For example, the *geom_phylopic*, implemented by the *ggimage* package, can be used to overlay silhouette images on the external layers to compare morphological characteristics with other attributes (e.g., taxonomy order, dietary preferences, and environmental variables) (fig. 1*D*). With this feature, *ggtreeExtra* supports more data types and visualization methods than other tools, since the *ggplot2* community has developed many geometric layers (fig. 1*B*; supplementary tables S1 and S2, Supplementary Material online). For instance, taxon-specific infographics can be added as insets in *ggtreeExtra* using the *geom_plot* layer provided by the *ggpmics* package (supplementary fig. S5*A*, Supplementary Material online). The *ggtreeExtra* package makes no assumption about user data. Given a suitable geometric layer, *ggtreeExtra* can incorporate and visualize any kind of information with a tree. This unique feature ensures the versatility of *ggtreeExtra*, making it easy to represent heterogeneous data from different disciplines.

A unique advantage of the circular layout is to create a chord diagram to reveal complex relationships. Couple with the inward circular tree layout supported by *ggtree* (Yu et al. 2017), *ggtreeExtra* allows displaying flows or connections between taxa, such as syntenic linkage among genes and genomes, and reticulate evolutionary relationships including horizontal gene transfer, hybridization, and interspecific recombination. This makes *ggtreeExtra* an ideal tool for exploring relationships or interactions between taxa in a compact way, and it is extremely powerful and uniquely suitable for microbiome research to present microbial correlation or interaction network with phylogenetic tree and other associated data. To demonstrate this unique feature, we used *ggtreeExtra* and *ggtree* (Yu et al. 2017) to integrate and visualize several data sets from *Arabidopsis* leaf microbiome (Bai et al. 2015) on the phylogenetic tree, including directional interactions among different bacteria strains, number of target genes, strain abundance, taxonomy information, and the biosynthetic potential of the isolates. The phylogenetic tree was visualized using an inward circular layout and the interaction data were visualized as a chord diagram connecting the corresponding isolates of the tree leaves. Other information was displayed as a stacked bar chart, heatmaps, and symbolic points on the tree (fig. 1*E*). With *ggtreeExtra* incorporating all the information, some of the evolutionary patterns that are not straightforward might become more obvious. For example, in figure 1*E*, we can easily find that the inhibitor interactions are more widely observed at strains from Firmicutes and Gammaproteobacteria, whereas strains from Alphaproteobacteria and Betaproteobacteria prefer sensitivity interactions. After the subsequent Mann−Whitney *U* test, the number of different interactions among these strains was confirmed to be significant (supplementary fig. S10, Supplementary Material online). To our knowledge, there are no other software tools that can easily produce such the figure, and the visualization indeed help us explore the data and generate new insights as our findings were not revealed in the original paper (Helfrich et al. 2018).

The *ggtreeExtra* is a subpackage of the *ggtree* package suite and takes all the advantages of other *ggtree* subpackages. Phylogenetic data imported by the *treeio* (Wang et al. 2020) package can be used in *ggtreeExtra*. This allows evolutionary inferences (e.g., clade support, molecular dating, and selection pressure) from commonly used software to be linked to other associated data (e.g., observational and experimental data) for integrative and comparative study (supplementary fig. S6, Supplementary Material online). Tree data can be processed using the *tidytree* package and a phylogenetic tree visualized by *ggtree* with fully annotation can be further annotated in *ggtreeExtra* with data layers especially in circular layout (supplementary figs. S5−S8, Supplementary Material online; fig. 1*E*). The *ggtreeExtra* package extends the capabilities of *ggtree* and fully supports the grammar of graphics implemented in *ggplot2* (Wickham 2016) (fig. 1*A*). It supports aesthetic mapping (supplementary figs. S3−S6, Supplementary Material online) and a layered grammar of graphics (supplementary figs. S7−S9, Supplementary Material online). Users can use scale functions to specify how the data were mapped to visual values and theme functions to adjust graphic appearance (supplementary figs. S3−S9, Supplementary Material online). Moreover, it takes all benefits of the *ggplot2* community. Geometric layers defined in *ggplot2* and other extension packages can be used in *ggtreeExtra* to visualize tree data (supplementary table S2 and figs. S2−S5, Supplementary Material online). We proposed and implemented this framework design originally in *ggtree* (Yu et al. 2018) and *ggtreeExtra* fully embraces the design concept. This is the beauty of the *ggtree* and *ggtreeExtra* and lays the foundation for displaying tree annotated data layers. It allows *ggtreeExtra* to support more visualization methods and has no assumption of the input data types (supplementary table S1 and S2, Supplementary Material online). As the *ggplot2* community keeps expanding, there will be more methods implemented which can be employed to create tree data layers in *ggtreeExtra*. Furthermore, the combination of these methods allows *ggtreeExtra* to create more possibilities than other tools to integrate more diverse data sets for novel exploratory data analysis (fig. 1*B* and *E*). Therefore, it has more potential to reveal systematic patterns and insights of our data than other tools. The versatility of this package ensures its applications in different research areas such as population genetics, molecular epidemiology and microbiome.

## Supplementary Material

Supplementary data are available at *Molecular Biology and Evolution* online.

## Supplementary Material

msab166_Supplementary_Data
