# Supplementary material for: ggtreeExtra: Compact Visualization of Richly Annotated Phylogenetic Data
Source: Mol Biol Evol. 2021 Jun 7;38(9):4039–42. doi: 10.1093/molbev/msab166 (PMC8382893; doi:10.1093/molbev/msab166)
Supplement: msab166_Supplementary_Data [file msab166_supplementary_data.pdf]

Supplemental File of

ggtreeExtra: Compact visualization of richly annotated phylogenetic data

Shuangbin Xu, Zehan Dai, Pingfan Guo, Xiaocong Fu, Shanshan Liu, Lang Zhou, Wenli Tang, Tingze Feng, Meijun Chen, Li Zhan, Tianzhi Wu, Erqiang Hu, Yong Jiang*, Xiaochen Bo* and Guangchuang Yu*

*correspondence: Guangchuang Yu <gcyu1@smu.edu.cn>, Xiaochen Bo <boxc@bmi.ac.cn> and Yong Jiang <jiang48231@163.com>

1 Overview of the *ggtreeExtra* package

Integrating and visualizing associated data with a phylogenetic tree help to identify biological patterns and generate new hypotheses. The associated data sets are diverse (*e.g.* abundance of species, gene expression level, taxonomy information of species, type and scale of target genes, and sampling information). Several tools have been developed to integrate and display associated data on a phylogenetic tree. However, only a few annotation layers and tree layouts are supported and most of the tools were designed for specific domains (Tab. S1). Here, we developed *ggtreeExtra* as a universal tool to annotate multi-dimensional data on the external panels of a phylogenetic tree (Fig. S1). It can link *ggtree* (Yu et al. 2017) and **geom** functions defined in *ggplot2* (Wickham 2016) or other ggplot2 extensions. It supports most of the tree layouts defined in *ggtree* (Yu et al. 2017). The tree can be annotated by **geom** functions defined in *ggtree* (Yu et al. 2017) before passing it to *ggtreeExtra* (Fig. S1). The *ggtreeExtra* package was developed based on the grammar of graphics (Wilkinson 2012) implemented in the ggplot2 package. Users can easily map variables (*e.g.* abundance of species, length of the genome, or sampling location) of associated data to aesthetic attributes (*e.g.* size, color, and shape) for visualizing external geometric objects (*e.g.* bar chart, point, or box plot) with a phylogenetic tree. The details (such as the actual mapping of visual values, figure legends, and plot appearance) of the graphics can be adjusted by the corresponding *scale* or *theme* functions defined in ggplot2 (Wickham 2016) (Fig. S1). Compared to other tools, *ggtreeExtra* supports more layouts and geometric layers for phylogenetic tree annotation. Making it more universal to handle data from different disciplines (Tab. S1 and Fig. S2).

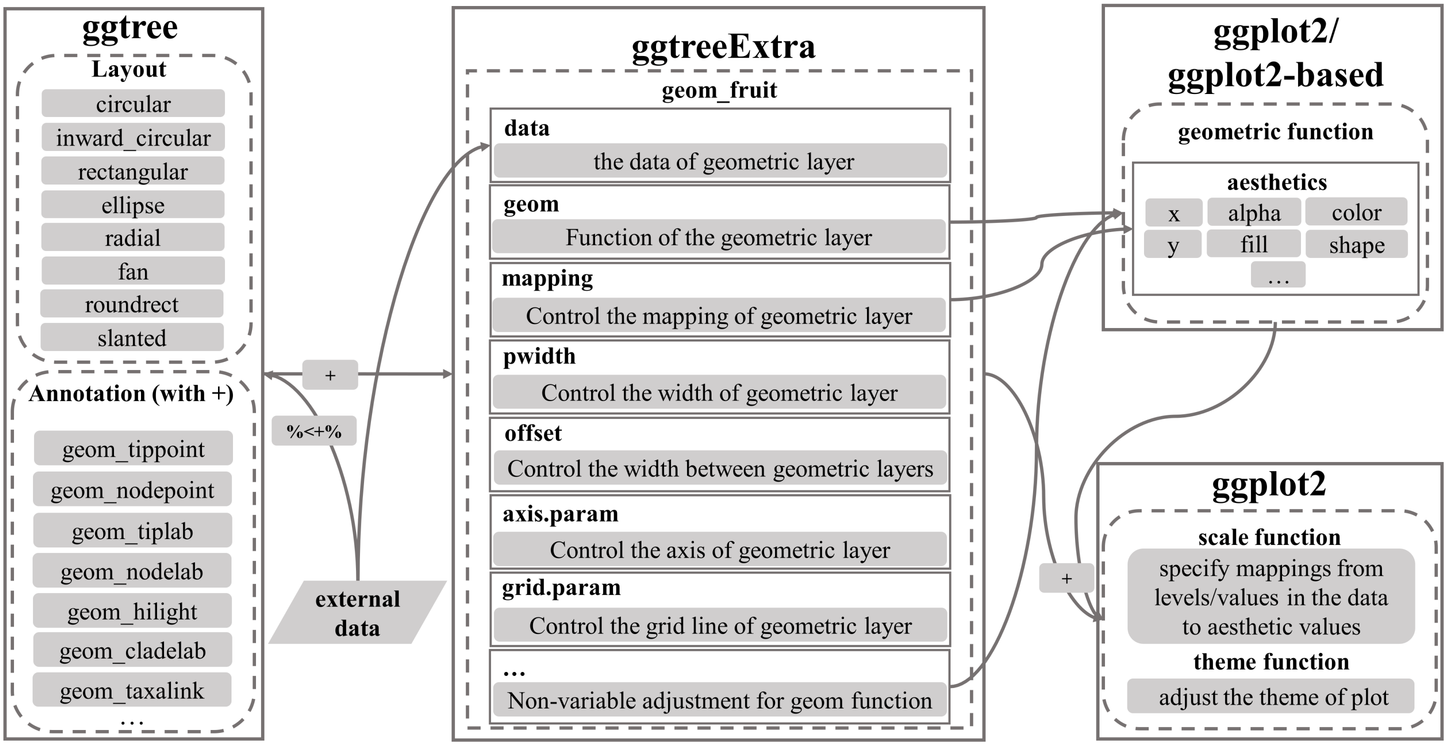

Fig. S1: **Overview of the design of *ggtreeExtra* package.**

Table S1: Comparing features in ggtreeExtra and other tools

Tool	Platform	Supported layouts for tree annotation[1]	Annotation layers	Layer combination[2]	Layer operation	Has a grammar	User interface
ggtreeExtra	R package	circular, inward circular, rectangular, slanted, ellipse, round rectangular, radial	Heatmap, scatter plot, simple bar chart, pattern bar chart, stacked bar chart, dodged bar chart, boxplot, pattern boxplot, violin plot, dot intervals plot, density plot, line plot, pie chart, image plot, MSA[3], inset plot	Yes	add, modify, delete	Yes	programming and command line
GraPhlAn	Python package	circular	Heatmap, scatter plot, simple bar chart	Yes	add	No	command line with config file
ETE3	Python package	circular, rectangular	Heatmap, scatter plot, simple bar chart, stacked bar chart, line plot, pie chart, image plot, inset plot, protein domain, MSA	Yes	add	No	programming and command line
iTOL	Web tool	circular, rectangular, slanted, ellipse, inward circular	Heatmap, scatter plot, simple bar chart, stacked bar chart, dodged bar chart, boxplot, line plot, pie chart, image plot, protein domain, MSA	Yes	add	No	interactive (mouse click with config file)
Microreact	Web tool	circular, rectangular	Heatmap, scatter plot	Yes	add	No	interactive (mouse click and command line with config file)
Evolview	Web tool	circular, rectangular, slanted, round rectangular	Heatmap, scatter plot, simple bar chart, stacked bar chart, protein domain	Yes	add	No	interactive (mouse click with config file)

[1] tree annotation: tree and data graphic alignment;
[2] Layer combination: whether layers listed in the 'Annotation layers' column can be combined freely;
[3] MSA: multiple sequence alignment

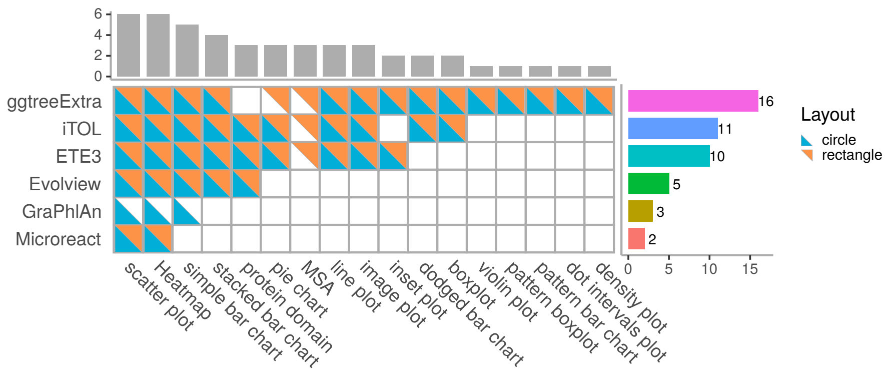

Fig. S2: **Visualization methods for tree annotation supported by different software tools.** *Note:* circle: circular, inward circular, radial; rectangle: rectangular, slanted, ellipse, round rectangular.

2 Installation

2.1 Local platform

To install `ggtreeExtra` package, please start R (version >= "4.0") and enter:

```
if (!requireNamespace("BiocManager", quietly = TRUE))
    install.packages("BiocManager")
BiocManager::install("ggtreeExtra")
```

To reproduce examples in this document, you need to install several extra packages.

```
# For Fig.S3
BiocManager::install(c("ggtree", "phyloseq"))
install.packages(c("ggplot2", "patchwork", "ggridges", "tibble", "dplyr"))

# For Fig.S4, you also need to install 'ggpattern'.
# Because ggpattern has not been released on 'CRAN',
# we need to install it from the github repository.

if (!requireNamespace("remotes", quietly = TRUE))
    install.packages("remotes")
remotes::install_github("coolbutuseless/ggpattern")
install.packages("tidyr")

# For Fig.S5

BiocManager::install("treeio")
install.packages(c("tidytree", "ggpmisc", "ggimage"))

# For Fig.S6
install.packages("ggnewscale")

# For Fig.S7
install.packages("ggstar")

# For Fig.S9
BiocManager::install("MicrobiotaProcess")
```

The source codes and datasets to produce this file can be obtained from the github repository, https://github.com/YuLab-SMU/plotting-tree-with-data-using-ggtreeExtra.

2.2 Docker image

We also provided a docker image[1] to help users to build the computing environment. Users can pull and run it according to the following commands. They don't need to install the dependency packages.

1. Install Docker (https://www.docker.com/). For example:

```
# Terminal of Ubuntu
sudo apt-get install docker.io
```

2. Pull the Docker image from Docker Hub:

```
# Terminal of Ubuntu
sudo docker pull xushuangbin/ggtreeextraarticleenv:latest
```

3. Run the image:

```
# Terminal of Ubuntu
sudo docker run -e PASSWORD=yourpassword -p 8787:8787 xushuangbin/ggtreeextraarticleenv
```

4. Log in to RStudio at `http://localhost:8787` using username `rstudio` and password `yourpassword`. For Windows users, you also need to provide your IP address, you can find it using `docker-machine ip default`. Inside the RStudio, run: `browseVignettes(package = "ggtreeExtraArticleEnv")`

5. You can click one of the links: "PDF", "source", "R code". In case of `The requested page was not found` error, try add 'help/' in front of the hostname in the URL (this is a known bug):

   `http://localhost:8787/help/library/ggtreeExtraArticleEnv/doc/supplementary_file.pdf`

[1] https://hub.docker.com/r/xushuangbin/ggtreeextraarticleenv

3 Geometric layers supported by *geom_fruit* of *ggtreeExtra*

The *ggtreeExtra* package was designed to link ggtree (Yu et al. 2017) and **geom** functions defined in *ggplot2* (Wickham 2016) and other *ggplot2* extension packages (Fig. S1). The *ggtreeExtra* package implemented a layer function, *geom_fruit*, which can internally reorder associated data based on the structure of a phylogenetic tree, visualize the data using specific geometric layer function with user-provided aesthetic mapping and non-variable setting, the graphic layer will be displayed on the external panel (right-hand side for rectangular layout and outer ring for circular layout). Here is the list of the geometric layer functions which work seamlessly with the *geom_fruit* function (Tab. S2).

Table S2: List of geometric layers supported by 'geom_fruit()'

**Package**	**Geom layer**	**Visual characteristic**	**Description**
	geom_dots	alpha, color, fill, size, shape	creates dotplots that automatically determines a bin width that ensures the plot fits within the available space
	geom_dotsinterval	alpha, color, fill, size, shape	creates dots, intervals, and quantile dotplots
ggdist	geom_pointinterval	alpha, color, fill, size, shape	creates point and multiple uncertainty interval
	geom_slab	alpha, color, fill	creates slab geom
	geom_slabinterval	alpha, color, fill	creates slab, point and interval meta-geom
ggimage	geom_image	alpha, color, size	visualizes image files
	geom_phylopic	alpha, color, size	queries image files from phylopic database and visualizes them
ggmsa	geom_msa	font,color,char_width	Multiple sequence alignment layer for ggplot2
	geom_bar_pattern	pattern_alpha, pattern_color, pattern_fill	draws bar charts with support for pattern fills
		pattern_angle, alpha, color, fill	
	geom_boxplot_pattern	pattern_alpha, pattern_color, pattern_fill	draws box and whiskers plot with support for pattern fills
		pattern_angle, alpha, color, fill	
ggpattern	geom_col_pattern	pattern_alpha, pattern_color, pattern_fill	draws bar charts using 'stat_identity()'
		pattern_angle, alpha, color, fill	with support for pattern fills
	geom_tile_pattern	pattern_alpha, pattern_color, pattern_fill	draws rectangle by using the center of the tile and its
		pattern_angle, alpha, color, fill	size with support for pattern fills
	geom_violin_pattern	pattern_alpha, pattern_color, pattern_fill	draws violin plot with support for pattern fills
	geom_bar	alpha, color, fill	draws bar charts
	geom_boxplot	alpha, color, fill	draws box and whiskers plot
	geom_col	alpha, color, fill	draws bar charts using 'stat_identity()'
	geom_label	alpha, color, fill, size	draws a rectangle behind the text
ggplot2	geom_point	alpha, color, fill, shape, size	creats scatterplots
	geom_raster	alpha, fill	a high performance special case for all the tiles are the same size
	geom_text	color, size	adds text to the plot
	geom_tile	alpha, color, fill	draws rectangle by using the center of the tile and its size
	geom_violin	alpha, color, fill	A violin plot is a compact display of a continuous distribution
ggpmisc	geom_plot	vp.width,vp.height	ggplot objects as insets to the base ggplot, using syntax similar to that of 'geom_label'
	geom_table	size	adds a textual table directly to the ggplot, using syntax similar to that of 'geom_label'
	geom_text_repel	color, size	adds text to the plot. The text labels repel away from each other and away from the data points
ggrepel	geom_label_repel	alpha, color, fill, size	draws a rectangle underneath the text. The text labels repel away
			from each other and away from the data ponts
	geom_density_ridges	alpha,fill	arranges multiple density plots in a staggered fashion
	geom_density_ridges2	alpha,fill	arranges multiple density plots in a staggered fashion
ggridges	geom_density_ridges_gradient	fill	draws multiple density plots with fill gradients along the x-axis
	geom_ridgeline	alpha,color,fill	plots the sum of the 'y' and 'height' aesthetics versus 'x', filling the area between 'y' and 'y + height' with a color
	geom_ridgeline_gradient	color,fill	works just like 'geom_ridgeline' except that the 'fill' aesthetic can vary along the x-axis
	geom_barh	alpha, color, fill	horizontal version of 'geom_bar()'
ggstance	geom_boxploth	alpha, color, fill	horizontal version of 'geom_boxplot()'
	geom_colh	alpha, color, fill	horizontal version of 'geom_col()'
ggstar	geom_star	alpha, color, fill, size, starshape	creates scatterplots
ggsymbol	geom_symbol	alpha, color, fill, size, symbolshape	creates scatterplots
ggtext	geom_richtext	alpha, color, fill, size, fontface	draws text labels similar to 'ggplot2::geom_label()',but formatted using basic markdown/html
scatterpie	geom_scatterpie	alpha, color, fill	creates scatter pie plot

Each geometric layer function has its own unique geometric attributes (Tab. S2). Users can choose appropriate geometric layer functions according to the type of input data. The variables of data can be mapped to visual attributes of the corresponding geometric layer function. For example, if users want to view the distribution and uncertainty (continuous data) of associated data in different groups (such as the gene expression or species abundance in different samples), several methods, including box plot, violin plot, and density plot, can be used (Fig. S3). For simple numeric data (*e.g.* length of the genome, the abundance of species), they can be visualized with a simple (pattern) bar plot or grouped (pattern) bar plot. Specifically,a pattern bar plot is efficient to visualize count data for different groups in grayscale (Fig. S4). The non-variable aesthetic setting is also supported.

To demonstrate the usage of *ggtreeExtra* with other *ggplot2*-based packages, we provided several examples, including density plot with *ggridges* (Fig. S3A and C), boxplot with *ggplot2* (Fig. S3B and D) and *ggpattern* (Figure S4C and D), bar chart with *ggpattern* (Fig. S4A and B), subplot with *ggpmisc* (Fig. S5A) and silhouette image with *ggimage* (Fig. S5B). These examples also illustrate the design of the *ggtreeExtra* package (Fig. S1). The *ggtreeExtra* package supports the grammar of graphic syntax and allows employing geometric layers defined in the *ggplot2* community to visualize phylogenetic data with a tree. With the development of the ggplot2 community, *ggtreeExtra* supports more geometric layers compared to other tools (Tab. S1 and Fig. S2). The latest version of *ggtreeExtra* can integrate heatmap, scatter plot, simple (grouped) (pattern) bar plot, simple (grouped) (pattern) box plot, violin, dot intervals plot, density plot, pie, image plot, inset plot (Tab. S1 and Fig. S2). Even though other tools might support similar graphic layers, *ggtreeExtra* is more powerful and flexible since it inherits all the features and advantages of the geometric functions developed by the community. For example, *iTOL* (Letunic and Bork 2019),

ETE3 (Huerta-Cepas, Serra, and Bork 2016), and *ggtreeExtra* all support displaying image plot on the phylogenetic tree, but the only *ggtreeExtra* supports mapping variable to color and scale the size of the images (Fig. S5B[2]). Besides, *ggtreeExtra* supports several visualization methods that cannot be found elsewhere. For example, subplots that describe statistical information of taxa can be embedded as insets in *ggtreeExtra* (Fig. S5A) to annotate corresponding taxa. As the *ggplot2* (Wickham 2016) community keeps expanding, there will be more *geom* functions implemented in either *ggplot2* (Wickham 2016) or other extensions and *geom_fruit* will gain more power to present data in the future.

3.1 Visualizing distribution of species abundance

Incorporating a phylogenetic tree with the information of species abundance is becoming increasingly common to investigate microbial communities. Here, a microbiome data provided in the *phyloseq* package (McMurdie and Holmes 2013) was used as an example. Density plot and box plot were used to display the abundance data via the specified *geom* (*i.e.*, *geom_density_ridge* and *geom_boxplot*). The abundance data (*melt_simple*) was a data frame that has a column of tip labels (*i.e.*, *OTU*) of the phylogenetic tree and another column of the species abundance (*i.e.*, *val*). The data frame was imported by the *data* argument of the *geom_fruit* function and visualized by *geom_density_ridges* and *geom_boxplot*. The *OTU* column (tip labels) was assigned to the *y* axis and the *val* column (abundance information) was assigned to the *x*-axis in the *aes* mapping. The phylum information (Phylum variable in the *ggtree* object, *p*) was assigned to the *fill* aesthetic mapping to color the density ridge plot and box plot (Fig. S3).

Visualizing data in *ggtreeExtra* is as simple as visualizing the data in *ggplot2*. The critical elements for a *ggplot2* graphic are the same – a data frame and a geometric layer to visualize the data with specific aesthetic mapping. Users don't need to care about how the tree was linked to the data, since the *geom_fruit* function will do all the data manipulation for us. We don't need to care about how the data value was mapped to the visual values either. Using aesthetic mapping by specifying a variable to a visual attribute (*e.g.*, map the *Phylum* variable to fill the colors of the density plot and boxplot) is directly supported in *ggtreeExtra* and users can use *scale* functions to specify how the values in the data were converted to the visual values (Fig. S3B and D).

```r
library(ggtree)
library(ggplot2)
library(ggtreeExtra)
library(patchwork)
library(ggridges)
library(phyloseq)

set.seed(1024)

data("GlobalPatterns")
GP <- GlobalPatterns

GP <- prune_taxa(taxa_sums(GP) > 1000, GP)

sample_data(GP)$human <- get_variable(GP, "SampleType") %in%
  c("Feces", "Skin")

mergedGP <- merge_samples(GP, "SampleType")
mergedGP <- rarefy_even_depth(mergedGP, rngseed=1024)
mergedGP <- tax_glom(mergedGP,"Order")

melt_simple <- psmelt(mergedGP) %>%
  dplyr::filter(Abundance < 120) %>%
  dplyr::select(OTU, val=Abundance)

p <- ggtree(mergedGP, size = 0.3) +
     geom_tippoint(
         mapping=aes(color = Phylum),
         show.legend = FALSE,
         size=0.6
     )
```

[2] The data that used to generate Fig. S5B is from Fig.1 of (Song et al. 2020) and it is available on https://github.com/tanaes/tetrapod_microbiome_analysis

```r
p1 <- p +
      geom_fruit(
          data = melt_simple,
          geom = geom_density_ridges,
          mapping = aes(
                        y = OTU,
                        x = val,
                        fill = Phylum
                   ),
          offset = 0.12,
          pwidth = 0.4,
          lwd = .05,
          axis.params = list(
                            axis = "x",
                            text.size  = 1,
                            hjust = 0.5,
                            vjust = 1
                        ),
          grid.params = list(),
          show.legend = FALSE
      )

p2 <- p +
      scale_color_viridis_d() +
      geom_fruit(
          data = melt_simple,
          geom = geom_boxplot,
          mapping = aes(
                        y = OTU,
                        x = val,
                        fill = Phylum
                   ),
          offset = 0.12,
          pwidth = 0.4,
          size = 0.1,
          outlier.size = 0.4,
          outlier.stroke = 0.06,
          outlier.shape = 21,
          axis.params = list(
                            axis = "x",
                            text.size = 1,
                            hjust = 0.5,
                            vjust = 1
                        ),
          grid.params = list(),
          show.legend = FALSE
      ) + scale_fill_viridis_d()

p3 <- p1 + layout_circular()

p4 <- p2 + layout_circular()

(p1 + p2)/(p3 + p4) + plot_annotation(tag_levels = 'A')
```

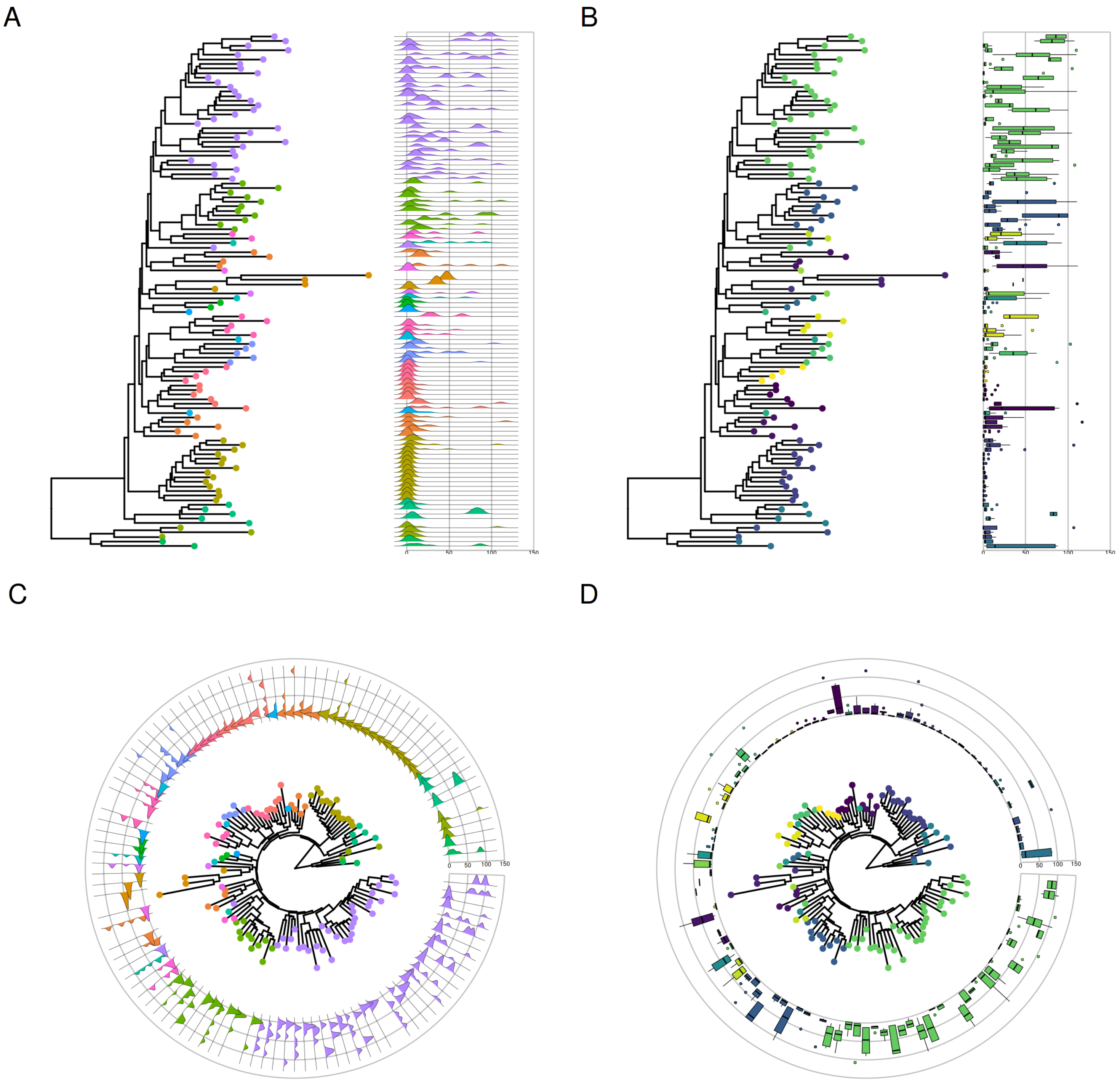

Fig. S3: **This example demonstrates how to use *ggtreeExtra* with *geom_density_ridges* layer from the *ggridges* package (Wilke 2020) and *geom_boxplot* layer from the *ggplot2* package (Wickham 2016)**. The associated data (*i.e.*, *melt_simple*) was imported by the *data* argument of the *geom_fruit* function. The data had a column (*i.e.*, *OTU*) of taxa labels which was mapped to the *y* axis. The *val* column contains the abundance of the species (continuous data) was mapped to the *x* axis and the *phylum* column contains phylum information of the species (categorical data) was mapped to the *fill* aesthetic to color the density ridge plot and box plot. This dataset was visualized by *facet_plot* function from *ggtree* (Fig.1. of (Yu et al. 2018)). However, it only supports rectangular layout (A) while *ggtreeExtra* supports more layouts and allows transformation between different layouts. In this example, subplot C and D were transformed from A and B.

3.2 Versatile bar and box plots by filling with colors and patterns

In this example, we used simulated data to demonstrate the usage of *ggtreeExtra* with the *ggpattern* package (FC 2020). We first simulated a random tree and two data frames with random values. Each data frame contains three columns of tip labels (*i.e.*, *id*), numerical data (*i.e.*, *value*), and grouping information (*group* or *class*). Visualizing data by bar and boxplot by filling with different patterns for different groups is quite common in the situation that grayscale figures are wanted. The *ggpattern*

package extends several *ggplot2* layers to support filling areas with geometric and image-based patterns.

Here we used *geom_bar_pattern* and *geom_boxplot_pattern* to visualize the simulated data with *ggtreeExtra* (Fig. S4). The phylogenetic tree was visualized by *ggtree* with different layouts (*e.g.*, rectangular, slanted, and fan). The data frame was imported by the *geom_fruit* function and was visualized as a bar chart by *geom_bar_pattern* or boxplot by *geom_boxplot_pattern* with the tree side by side. Data variables were mapped to visual attributes (*e.g.*, *id* for the y-axis, *value* for the x-axis, and *group* for pattern), including layer-specific mapping (*e.g.*, *pattern*, *pattern_fill*, and *pattern_angle*). Non-variable adjustments were allowed to pass to the *geom* function within *geom_fruit* (*e.g.*, pattern spacing and size). The *ggtreeExtra* also supports setting background grid line (Fig. S3) and axis label (Fig. S4).

```
library(ggtree)
library(ggtreeExtra)
library(ggpattern)
library(ggplot2)
library(patchwork)

set.seed(1024)
tr <- rtree(20)
dat <- tibble::tribble(
            ~value, ~group,
            abs(rnorm(5, 10, sd = 3)), "A",
            abs(rnorm(3, 12, sd = 2)), "B",
            abs(rnorm(5, 9, sd = 3)),  "C",
            abs(rnorm(7, 6, sd = 2)),  "D"
       ) %>% tidyr::unnest(value)
dat$id <- tr$tip.label
dt <- tibble::tribble(
           ~value, ~class,
           abs(rnorm(40, 10, sd = 3)), "A",
           abs(rnorm(24, 12, sd = 2)), "B",
           abs(rnorm(40, 9, sd = 3)), "C",
           abs(rnorm(56, 6, sd = 2)), "D"
      ) %>% tidyr::unnest(value)
dt$id <- c(rep(tr$tip.label[1:5], 8), rep(tr$tip.label[6:8], 8),
           rep(tr$tip.label[9:13],8), rep(tr$tip.label[14:20], 8)
          )

p1 <- ggtree(tr, size=0.2, branch.length="none")
p2 <- ggtree(tr, size=0.2, layout="slanted", branch.length="none")
p3 <- ggtree(tr, size=0.2, layout="fan", open.angle=180, branch.length="none")
p4 <- ggtree(tr, size=0.2, layout="fan", open.angle=180, branch.length="none")

p1 <- p1 +
      geom_fruit(
          data=dat,
          geom=geom_col_pattern,
          mapping=aes(y=id, x=value, pattern=group, pattern_angle=group),
          width=0.6,
          pwidth = 0.6,
          pattern_spacing =0.01,
          pattern_size = 0.1,
          pattern_density = 0.4,
          fill = "grey",
          pattern_fill="grey35",
          position=position_identityx(),
          axis.params=list(axis="x", text.size=1.2, hjust=0.5, vjust=1)
      ) +
      theme(legend.key.size = unit(0.3, 'cm'))

p2 <- p2 +
      geom_fruit(
```

```
        data=dat,
        geom=geom_col_pattern,
        mapping=aes(y=id, x=value, pattern=group, pattern_fill=group),
        width=0.6,
        pwidth = 0.6,
        pattern_spacing =0.01,
        pattern_size = 0.1,
        pattern_density = 0.4,
        fill = "grey",
        position=position_identityx(),
        axis.params=list(axis="x",text.size=1.2, hjust=0.5, vjust=1)
    ) +
    theme(legend.key.size = unit(0.3, 'cm'))

p3 <- p3 +
    geom_fruit(
        data=dt,
        geom=geom_boxplot_pattern,
        mapping=aes(y=id, x=value, pattern=class, pattern_angle = class),
        size=0.1,
        outlier.shape=NA,
        pwidth=0.5,
        pattern_size = 0.1,
        pattern_density = 0.4,
        pattern_spacing =0.01,
        fill = "grey",
        pattern_fill="grey35",
        position=position_dodgex(),
        grid.params=list(),
        axis.params=list(axis="x", text.size=1.2, hjust=0.5, vjust=1)
    ) +
    theme(legend.key.size = unit(0.35, 'cm'))

p4 <- p4 +
    geom_fruit(
        data=dt,
        geom=geom_boxplot_pattern,
        mapping=aes(y = id, x = value, pattern = class, pattern_fill = class),
        size = 0.1,
        outlier.shape = NA,
        pwidth = 0.5,
        pattern_size = 0.1,
        pattern_density = 0.4,
        pattern_spacing = 0.01,
        fill = "grey",
        position = position_dodgex(),
        grid.params = list(),
        axis.params = list(axis="x", text.size=1.2, hjust=0.5,  vjust=1)
    ) +
    theme(legend.key.size = unit(0.35, 'cm'))

(p1 + p2)/(p3 + p4) + plot_annotation(tag_levels = 'A')
```

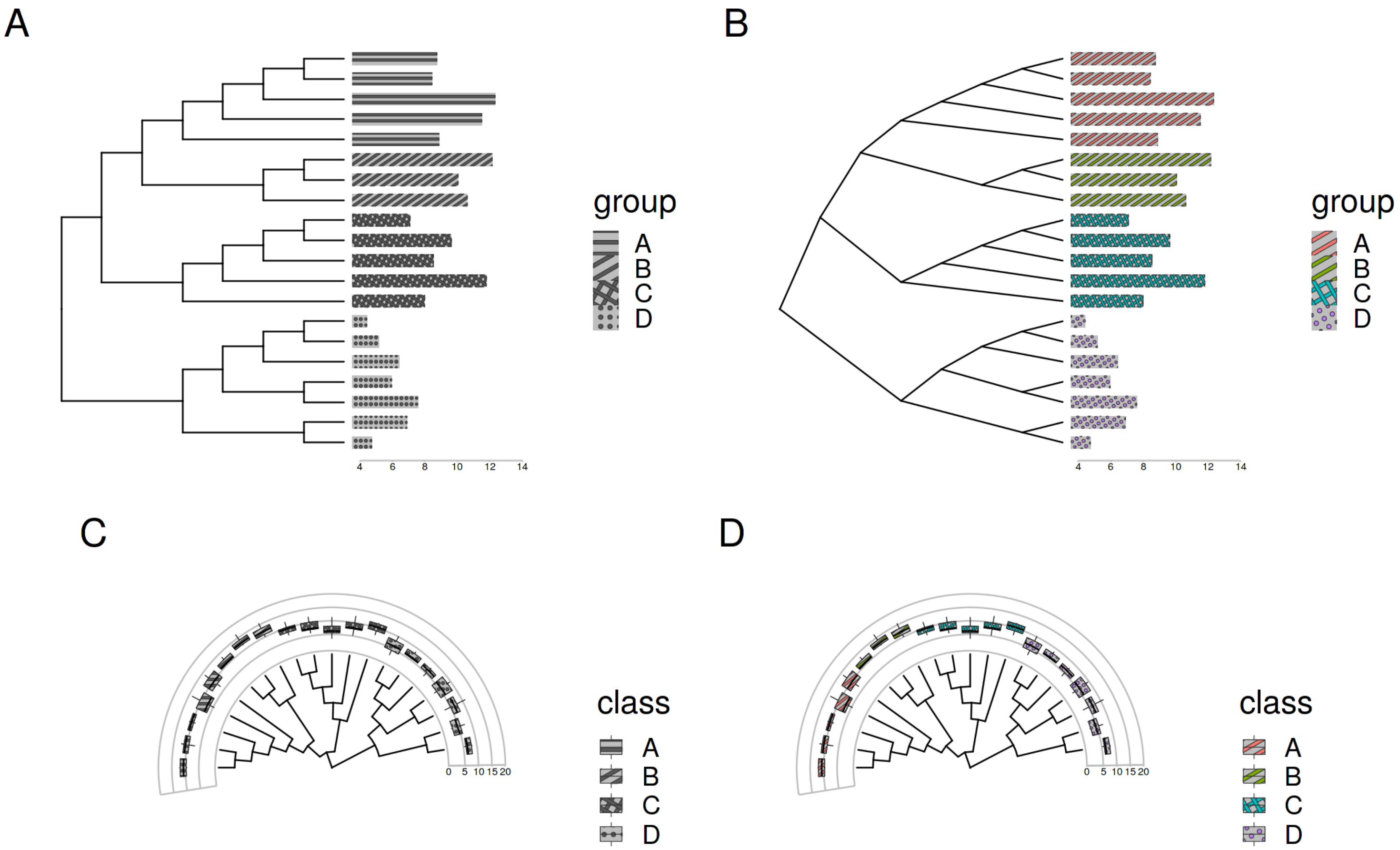

Fig. S4: **This example demonstrates using *ggtreeExtra* with *geom_bar_pattern* and *geom_boxplot_pattern* layers from the *ggpattern* package (FC 2020)**. Different tree layouts including rectangular (A), slanted (B), and fan (C and D) layouts are all supported in *ggtreeExtra*.

3.3 Taxon-specific graphical information

Displaying graphs of representative species on a phylogenetic tree is commonly used to compare morphological differences. ETE3 (Huerta-Cepas, Serra, and Bork 2016) and iTOL (Letunic and Bork 2019) support linking external images to any tree node. Besides linking external images, *ggtreeExtra* supports querying silhouette images directly from the PhyloPic database[3] and allows aesthetic mapping to fill the color and rescale the size of the images using tree-associated data variable. Moreover, with the aids of the *ggpmisc* package (Aphalo 2020), subplots that describe taxon-specific statistical information can be embedded as insets in *ggtreeExtra*.

In the first example (Fig. S5A), we simulated a tree with associated data of two taxa. The data contains numerical values for two different groups (*e.g*, species abundance for different samples or body mass index in different regions). The data of each taxon was visualized as a boxplot using *ggplot2*. The boxplots (complex *ggplot* objects) and the taxon labels were stored as a *tibble* object (similar to a data frame with the ability to store a list of arbitrary objects in a column). The *geom_fruit* function utilizes the *geom_plot* provided by the *ggpmisc* package to redraw the boxplots as insets and aligns the insets to corresponding taxa. This example also illustrates that *ggtreeExtra* allows using complex data as input if and only if there is a geometric layer supporting it.

The second example (Fig. S5B) reproduces Fig. 1 of (Song et al. 2020)[4]. The host tree was obtained from TimeTree (Kumar et al. 2017). The branch color represents the Mantel Pearson correlation of gut microbiome. The first ring uses a stack bar chart to represent the diet composition of the host. The second ring represents the host taxonomic class and the third ring represents flight status. Some of the representative species are displayed in the outermost ring and colored by taxonomic class using *geom_phylopic* provided by the *ggimage* package developed in-house. Providing the UID of PhyloPic for corresponding species, the *geom_phylopic* will automatically read the PhyloPic image directly from the PhyloPic database online and users don't need to download the images manually. The *ggimage* package also provides a function, *phylopic_uid*, to convert scientific names of species to PhyloPic UID and there is no need to search UID manually. This example demonstrated that using a variable (*e.g.* taxonomic class) to color the images is supported and this is a unique feature that is available in *ggtreeExtra*

[3]http://phylopic.org/
[4]data available on https://github.com/tanaes/tetrapod_microbiome_analysis

with the help of *ggimage*.

Either images or insets are simple layers and they can be combined with other layers supported in *ggtreeExtra*. In the example, bar chart, heatmap, and PhyloPic images are combined with the tree to generate Fig. S5B. More details and examples of visualizing multiple associated data using different geometric layers in *ggtreeExtra* will be introduced in the next session.

```
library(ggtreeExtra)
library(ggtree)
library(treeio)
library(tidytree)
library(ggnewscale)
library(ggpmisc)
library(ggplot2)
library(ggimage)
library(patchwork)

set.seed(1024)
tr <- rtree(30)

dat1 <- data.frame(value=c(abs(rnorm(10, 3)),abs(rnorm(10, 5))), group=c(rep("A",10),rep("B",10)))
dat2 <- data.frame(value=c(abs(rnorm(15, 6)), abs(rnorm(15, 3))), group=c(rep("A",15),rep("B",15)))

subp1 <- ggplot(dat1) +
         geom_boxplot(aes(x=group, y=value, fill=group),
                      size=0.1, outlier.size = 0.1, show.legend=F) +
         theme_bw() +
         theme(legend.key.size = unit(1, 'mm'),
               plot.background = element_rect(fill=NA, color=NA),
               panel.border = element_rect(size=0.1),
               legend.title = element_text(size=3.6),
               legend.text = element_text(size=3.2),
               axis.ticks = element_line(size=0.1),
               axis.text = element_text(size=2.8),
               axis.title = element_text(size=3))

subp2 <- ggplot(dat2) +
         geom_boxplot(aes(x=group, y=value, fill=group),
         size=0.1, outlier.size = 0.1, show.legend=F) +
         theme_bw() +
         theme(legend.key.size = unit(1, 'mm'),
               plot.background = element_rect(fill=NA, color=NA),
               panel.border = element_rect(size=0.1),
               legend.title = element_text(size=3.6),
               legend.text = element_text(size=3.2),
               axis.ticks = element_line(size=0.1),
               axis.text = element_text(size=2.8),
               axis.title = element_text(size=3))

dt <- tibble::tibble(id=c("t14", "t2"), plot=list(subp1, subp2))

p1 <- ggtree(tr,
             layout="fan",
             open.angle=0
             ) +
      geom_tiplab(align=T, size=2)

p2 <- p1 +
      geom_fruit(
                 data=dt,
                 geom=geom_plot,
                 mapping=aes(
```

```
                        y=id,
                        label=plot
                    ),
            offset=0.4,
            position=position_identityx(),
            vjust=0.4,
            hjust=0.7,
            vp.width=0.18,
            vp.height=0.22
      )

## subplot B
tr <- read.tree("../data/VertebrateGutMicrobiomes/annotated_host_tree.tre")
corda <- read.csv("../data/VertebrateGutMicrobiomes/mantel.jaccard.pearson.csv")
corda$r <- abs(corda$r)
barda <- read.csv("../data/VertebrateGutMicrobiomes/data_diet_bar.csv", check.names=F)
barda <- reshape2::melt(barda, id.vars="ID", variable.name="Diet", value.name="mete")
barda$Diet <- factor(barda$Diet, levels=c("Fruit","Invertebrates",
                                          "Nectar","Plants","Scavenging",
                                          "Seeds","Meat (Ectotherms)",
                                          "Meat (Endotherms)",
                                          "Meat (Fish)","Meat (Unknown)"))

cladeda <- read.csv("../data/VertebrateGutMicrobiomes/data_clade_class.csv", check.names=F)
cladeda$id <- nodeid(tr, cladeda$id)
cladeda$class <- factor(cladeda$class, levels=c("Amphibia","Chelonia","Lepidosauria",
                                                "Crocodylomorpha","Aves","Mammalia"))

flightda <- read.csv("../data/VertebrateGutMicrobiomes/data_flight_bar.csv")

phylopicda <- read.csv("../data/VertebrateGutMicrobiomes/data_phylopic_uid.csv")
phylopicda$class <- factor(phylopicda$class, levels=c("Amphibia","Chelonia","Lepidosauria",
                                                      "Aves","Mammalia"))

fig <- ggtree(tr, layout="fan", open.angle=5, continuous="color")

# Optional for color branches.
fig <- fig %<+% corda
fig$data$width <- ifelse(is.na(fig$data$r), 0.05, 0.4)
r <- NULL
fig <- fig +
     aes(color=r, size=I(width)) +
     scale_colour_viridis_c(
         name="Mantel Correlation",
         option="C",
         guide=guide_colorbar(
                   barheight = 0.35,
                   barwidth = 3.8,
                   order = 4,
                   title.position = "top",
                   label.position = "bottom",
                   direction = "horizontal")
     )
# Bar chart in the innermost ring
fig1 <- fig +
      geom_fruit(
          data=barda,
          geom=geom_bar,
          mapping=aes(x=mete, y=ID, fill=Diet),
          orientation="y",
```

```
          stat="identity",
          colour=NA,
          pwidth=0.25,
          offset=0.008,
          axis.params=list(axis="x", text.size=0.4)
      ) +
      scale_fill_manual(
          values=c("#a6cee3","#cab2d6",
                  "#1f78b4","#33a02c",
                  "#6a3d9a","#b2df8a",
                  "#fb9a99","#e31a1c",
                  "#ff7f00","#fdbf6f"),
          guide=guide_legend(keywidth=0.3, keyheight=0.3, order=1)
      )
# Clade label in the inner ring
fig2 <- fig1 +
      new_scale_colour() +
      geom_cladelab(
          data=cladeda,
          mapping=aes(node=id, label=class, colour=class),
          textcolour=NA,
          barsize=3,
          extend=0.2,
          offset=115) +
      scale_colour_manual(
          name="Host Class",
          values=c("#b2df8a","#33a02c","#fb9a99",
                   "#e31a1c","#EACB47","#6a3d9a"),
          guide=guide_legend(
                    keywidth=0.3,
                    keyheight=0.3,
                    order=2,
                    override.aes=list(size=1.5, alpha=1))
      )
# Clade in the middle ring
fig3 <- fig2 +
      new_scale_fill() +
      geom_fruit(
          data=flightda,
          geom=geom_tile,
          mapping=aes(y=ID, fill=flight),
          size=0,
          width=14,
          offset=0.18,
          pwidth=0.4
      ) +
      scale_fill_manual(
          name="Flight Status",
          values=c("black", "white"),
          guide=guide_legend(keywidth=0.3, keyheight=0.3, order=3,
                             override.aes=list(color="black", size=0.3))
      )
# Silhouettes image in the outermost ring
fig4 <- fig3 +
      new_scale_colour() +
      geom_fruit(
          data=phylopicda,
          geom=geom_phylopic,
          mapping=aes(y=taxa, image=uid, color=class),
          size=0.035,
```

```
        offset=0.16,
        alpha=0.8,
        position=position_identityx()
    ) +
    scale_colour_manual(
        values=c("#b2df8a","#33a02c","#fb9a99",
                 "#EACB47","#6a3d9a"),
        guide="none"
    ) +
    theme(
        legend.background=element_rect(fill=NA),
        legend.title=element_text(size=7),
        legend.text=element_text(size=5),
        legend.spacing.y = unit(0.02, "cm")
    )
p2 + fig4 + plot_annotation(tag_levels = 'A')
```

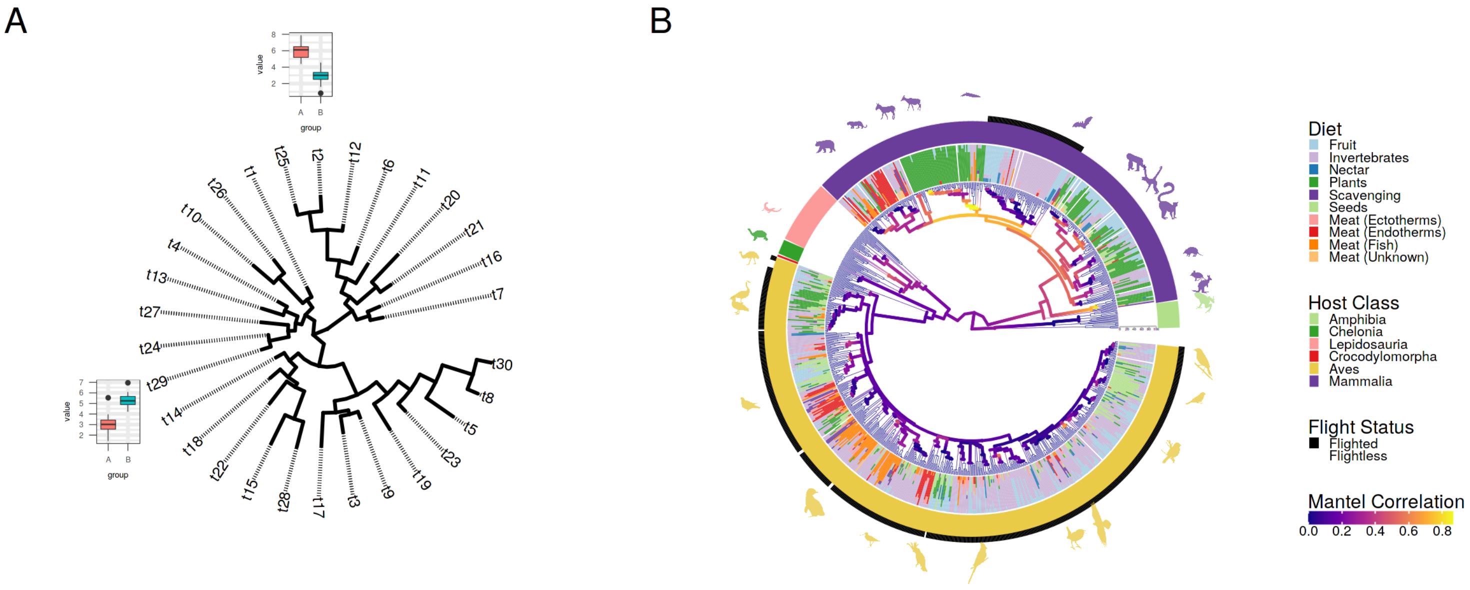

Fig. S5: **Using subplots and images as insets on a phylogenetic tree in *ggtreeExtra*.** Subplots can be a *ggplot* object generated by ggplot2 or its extensions that summarizes taxon-specific information (A). Taxon information can be used to color or scale the size of silhouette images (B).

4 Mapping and visualizing associated data using the *ggtreeExtra* package

The *ggtreeExtra* package allows different geometric layers defined in *ggplot2* (Wickham 2016) or ggplot2-extension packages (Tab. S1, S2, and Fig. S2) to be integrated on a phylogenetic tree. The combination of these geometric layers allows a number of associated data sets to be visualized with a tree side by side (Fig. S3 and S4). The *ggtreeExtra* package supports tree annotation for multiple tree layouts (Fig. S3, S4, S2, and Tab. S1), including rectangular and circular layouts. Circular layout is an efficient way to visualize multi-dimensional data sets since it can reduce space and make the graph more compact.

The associated data can be imported with the *data* parameter of the *geom_fruit* function (Fig. S3 and S4). The *geom_fruit* function can utilize tree data integrated to the ggtree graphic object by the `%<+%` operator (Yu et al. 2018) defined in the *ggtree* package (Fig. S1). In addition, tree data parsed by the *treeio* package (Wang et al. 2020) can also be used in *ggtreeExtra*.

The *ggtreeExtra* package was developed based on the grammar of graphics (Wilkinson 2012). It allows users to map variables (*e.g.* abundance of species, length of the genome, and sampling location) of the associated data to the visual attributes (*e.g.* color, size, shape) of the geometric objects (*e.g.* bar chart, point, boxplot) on the rectangular or circular phylogenetic tree (Fig. S1). Users are free to combine different geometric layers to create complex tree annotation. We present many examples to elucidate how to use *ggtree* and *ggtreeExtra* to visualize data on phylogenetic trees in the online book[5].

[5]http://yulab-smu.top/treedata-book

4.1 Data importation

The *ggtreeExtra* is a sub-package of the *ggtree* package suite and it works seamlessly with *ggtree* (Yu et al. 2017), *treeio* (Wang et al. 2020), and *tidytree*. Tree data parsed by *treeio* (Wang et al. 2020) can be used directly in *ggtreeExtra*. This allows evolutionary statistics inferred by commonly used software, such as *BEAST* (Drummond and Rambaut 2007), *RAxML* (Stamatakis 2014), *HyPhy* (Pond, Frost, and Muse 2005), *PAML* (Yang 2007), *ASTRAL* (Mirarab and Warnow 2015), *pplacer* (Matsen, Kodner, and Armbrust 2010), *RevBayes* (Höhna et al. 2016), PHYLODOG (Boussau et al. 2013) and EPA (Berger, Krompass, and Stamatakis 2011), to be incorporated and visualized with *ggtreeExtra*. Any data frame that contains a column of tip labels can be integrated into a *ggtree* graphic object using *%<+%* operator (Yu et al. 2018) and can be imported directly through the *geom_fruit*. Consequently, *ggtreeExtra* supports visualizing evolutionary statistics inferred by commonly used software tools (via the *treeio* package) and external associated data (*e.g.* experimental or clinical data) (Fig. S6). These unique features ensure that *ggtreeExtra* supports more diverse sources of data compared to other tools.

In previous examples (Figure S3-5), we have demonstrated the usage of importing external data directly in *geom_fruit*. Here, we reproduce Figure 3.3 (also served as the book cover) of (Smith and Wrighton 2019)[6] to show how to use internal data stored in a tree object and integrate external associated data to a tree using the `%<+%` operator and visualize the data in *ggtreeExtra*. The tree was built using *RAxML* (Stamatakis 2014) and was parsed by the *read.raxml* function provided by the *treeio* package (Wang et al. 2020). Clade support (*i.e.*, bootstrap values) will be parsed and stored in the output tree object. The tree was visualized by *ggtree* (Yu et al. 2017). There are associated data sets that contain the information of Ecosystem type, sequencing type, and sample treatment method (all categorical data). The first column of external data should be tip labels and the element of the column must be unique so that the data can be linked to the *ggtree* graphic object using the `%<+%` operator. The *geom_fruit* function can utilize internal tree data from the tree object (either parsed by *treeio* or integrated by *ggtree*). The variable of the data can be mapped to related attributes of the geometry layer. In this case of using internal data, the *y* aesthetic mapping can be ignored. Here we used heatmaps to visualize categorical data. The external ring heatmaps represent different types of corresponding categories (*i.e.*, the types of Ecosystem were mapped to the color of the innermost ring heatmaps, the types of sequencing methods were mapped to the color of the middle ring heatmap and the types of sample treatment were mapped to the color of the outermost ring heatmaps) (Fig. S6).

Comparing with other tools, there is no restriction of data types in *geom_fruit* of the *ggtreeExtra* package. It depends on the data types required by corresponding geometric functions (Tab. S2). For example, visualizing the distribution of species abundance (numerical data with unequal lengths) with a tree is not supported by other tools. Such data can be visualized as density plots using *geom_density_ridges* (Wilke 2020) in *ggtreeExtra* (Fig. S3A and C). Even *ggplot* graphic objects (a complex data structure produced by *ggplot2*) can be served as taxon-specific data and can be used in *ggtreeExtra* via the *geom_plot* layer provided by the *ggpmisc* package (Aphalo 2020). As long as a data type is supported by a geometric layer that works with *ggtreeExtra* (Tab. S2), it is supported by *ggtreeExtra*. Since there will more ggplot2 extensions developed in the future, more data types will be supported and the applications of *ggtreeExtra* in multi-discipline areas will also be expanded.

```r
library(ggtreeExtra)
library(ggtree)
library(treeio)
library(ggplot2)
library(ggnewscale)
library(tidytree)
tr <- read.raxml("../data/Methanotroph/Methanotroph_rpS3_Modified_Alignment_RAxML")
# Optional, Root the tree to the archaea sequences
treeda <- root(tr, node=1402, edgelabel=TRUE)
root <- rootnode(treeda)
# read associated data
df <- read.csv("../data/Methanotroph/metadata.csv")
# reset the levels of columns to reproduce the order of original figure.
df$Specific.Ecosystem <- factor(df$Specific.Ecosystem,
                                levels=c("Agriculture", "Alkaline/Hypersaline",
                                         "Contaminated/Wastewater", "Endosymbiont",
                                         "Freshwater", "Forest", "Geothermal",
                                         "Marine", "Natural Seep", "Peat",
                                         "Permafrost", "Wetland", "Unknown"))
df$MetaType <- factor(df$MetaType,
                      levels=c("Metatranscriptome", "Metagenome",
                               "Single-amplified genome", "Fosmid", NA))
df$Treatment <- factor(df$Treatment, levels=c("Native", "Enrichment","Isolate", "Unknown"))
```

[6]the source data is available on https://github.com/TheWrightonLab/Methanotroph_rpS3Analyses_SmithWrighton2018

```
p <- ggtree(treeda, layout="fan", open.angle=30)
print(as.treedata(p))
```

```
## 'treedata' S4 object'.
##
## ...@ phylo:
## Phylogenetic tree with 727 tips and 725 internal nodes.
##
## Tip labels:
##   3300019787==Ga0182031_12339262, 3300014838==Ga0182030_1009273110, gb_PLVF01000413_pos14750To15429=Methyloc
## Node labels:
##   , NA, NA, NA, NA, NA, ...
##
## Unrooted; includes branch lengths.
##
## with the following features available:
##  'bootstrap'.
```

```
p <- rotate_tree(p, 90)
p1 <- p +
      geom_treescale(x=0.2, y=727*6/11, width=1, offset=20) +
      geom_point2(aes(subset = !isTip & node != root,
                      fill = cut(bootstrap, c(0, 70, 90, 100), right = F)),
                  shape=21, size=1.2, stroke=0.3) +
      scale_fill_manual(values = c("black", "grey", "white"),
                        name = "Bootstrap (BP)",
                        breaks = c('[90,100)', '[70,90)', '[0,70)'),
                        labels = expression(BP >= 90, 70<=BP*'<90', BP<70),
                        guide=guide_legend(keywidth=0.5, keyheight=0.6,
                                           override.aes=list(size=2.5, stroke=0.3),
                                           order=1)
                        )
# we can use %<+% to integrate the external datasets to tree structure.
# and the y can not be specified in geom_fruit.
p2 <- p1 %<+% df +
      new_scale_fill() +
      geom_fruit(
          geom=geom_tile,
          mapping=aes(fill=Specific.Ecosystem),
          offset=0.13,
          width=0.35,
          axis.params=list(
                           axis="x", text="Ecosystem",
                           text.angle=0, hjust=0, text.size=3,
                           family="Times", fontface="bold"
                      )
      ) +
      scale_fill_manual(
          values=c("green3","turquoise", "maroon", "orchid",
           "deepskyblue", "forestgreen","salmon", "cadetblue3",
           "slategray4",  "yellowgreen", "gray90", "chocolate2",
           "yellow"),
          guide=guide_legend(title="Ecosystem", keywidth=0.5, keyheight=0.5, order=4),
          na.translate=FALSE
      )

p3 <- p2 +
      new_scale_fill() +
      geom_fruit(
          geom=geom_tile,
```

```r
          mapping=aes(fill=MetaType),
          offset=0.13,
          width=0.35,
          axis.params=list(
                          axis="x", text="Sequencing Type",
                          text.angle=0, hjust=0, text.size=3,
                          family="Times", fontface="bold"
                     )
      ) +
      scale_fill_manual(
          values=c("red", "black", "dodgerblue", "gray50"),
          guide=guide_legend(title="Sequencing Type", keywidth=0.5, keyheight=0.5, order=3),
          na.translate=FALSE
      )

p4 <- p3 +
      new_scale_fill() +
      geom_fruit(
          geom=geom_tile,
          mapping=aes(fill=Treatment),
          offset=0.13,
          width=0.35,
          axis.params=list(
                          axis="x", text="Sample Treatment",
                          text.angle=0, hjust=0,
                          text.size=3, family="Times", fontface="bold"
                     )
      ) +
      scale_fill_manual(
          values=c("red","gray50", "black", "yellow"),
          guide=guide_legend(title="Sample Treatment", keywidth=0.5, keyheight=0.5, order=2),
          na.translate=FALSE
      ) +
      theme(
            legend.background=element_rect(fill=NA), # the background of legend.
            legend.title=element_text(size=9, family="Times", face="bold"),
            legend.text=element_text(size=7, family="Times"), # the text size of legend.
            legend.spacing.y = unit(0.02, "cm"),
            legend.margin=margin(0.1, 0.9, 0.1,-0.9, unit="cm"), # t, r, b, l, cm
            legend.box.margin=margin(0.1, 0.9, 0.1, -0.9, unit="cm"),
            plot.margin = unit(c(-1.2, -1.2, -1.2, 0.1),"cm")
      )

# optional
cladeda <- data.frame(nodeid = c(793, 791, 1384, 1394, 1440, 1405),
                      label = c("Gammaproteobacteria", "Alphaproteobacteria",
                                "Ca.Methylomirabilis", "Methylacidiphilae",
                                "ANME-1", "ANME-2"),
                      horizontal = c(FALSE, FALSE, TRUE, TRUE, TRUE, TRUE),
                      hjust = c(0.5, 0.5, 0, 0, 0, 0))

p5 <- p4 +
      geom_cladelab(
          data = cladeda,
          mapping = aes(node=nodeid, label=label, horizontal=horizontal, hjust=hjust),
          angle = "auto", offset = 1.4,
          align = T, fontsize = 2,
          barsize = 1, family = "Times",
          fontface="bold", offset.text = 0.1
      )
```

```
p5
```

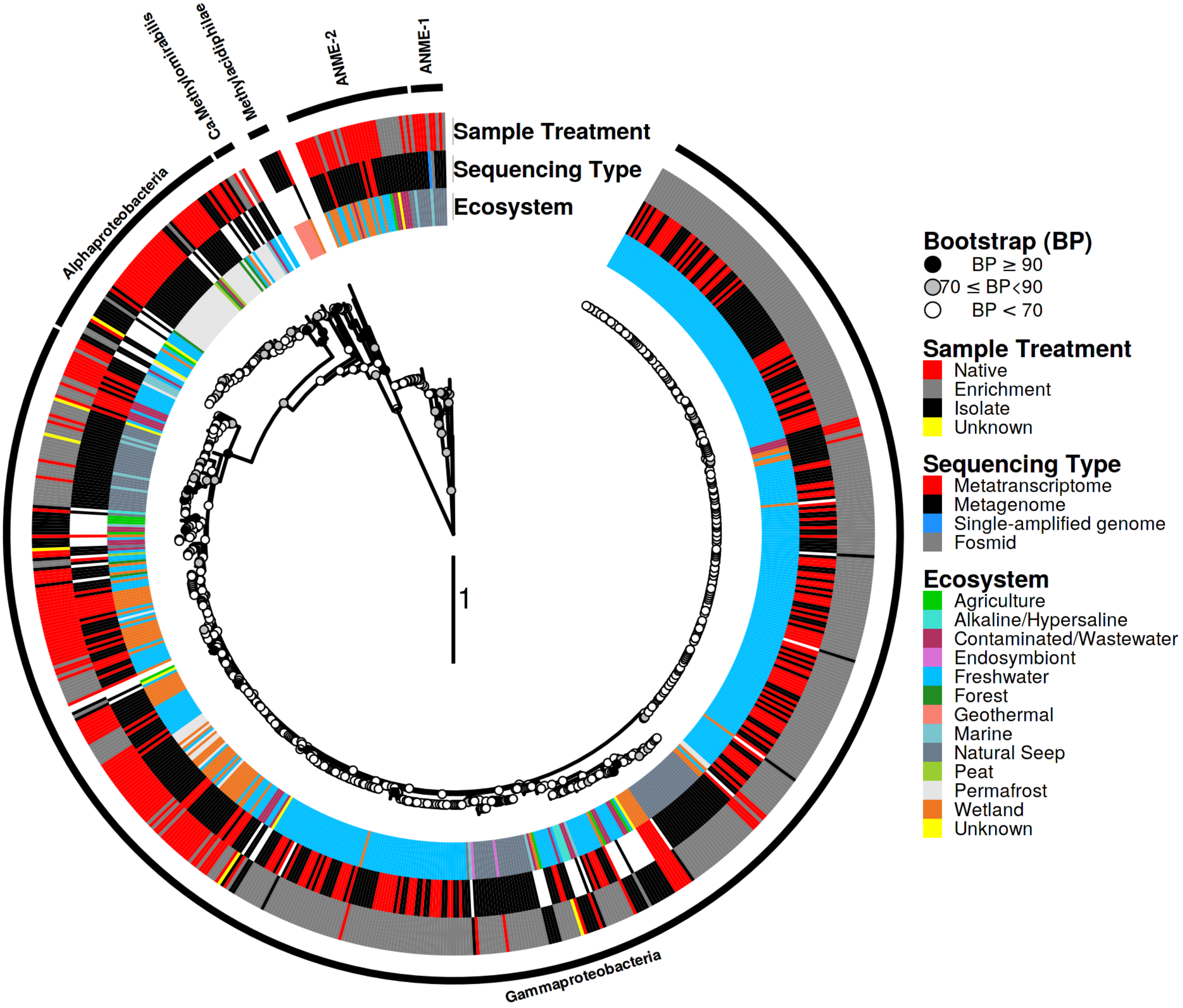

Fig. S6: **Phylogeny of methanotroph ribosomal protein S39 (rpS3) genes from Figure 3.3 of (Smith and Wrighton 2019)**. Bootstrap value was parsed with the tree by the *read.raxml* function and other associated data sets were integrated into the tree via the `%<+%` operator. The *ggtreeExtra* can directly use these data sets to visualize data layers.

4.2 Displaying multiple associated data using a layered grammar of graphics

The *ggtreeExtra* supports the grammar of graphics (Wilkinson 2012) implemented in the *ggplot2* package (Wickham 2016). In previous sessions, we have demonstrated the usages of aesthetic mapping, which allows users to map variables of associated data to visual characteristics at a high level of abstraction (Fig. S3, S4, S5, and S6). Other tools (Tab. S1), such as GraPhlAn (Asnicar et al. 2015), require users to convert associated data to visual values (*e.g.* color, position) manually. For example, the input for GraPhlAn is a phyloxml file which contains tree, visual values of associated data and configuration setting. Such data file is hard to prepare for ordinary users without programming expertise.

In this session, we emphasize another part of the grammar of graphics – the ggplot2 layered graphics. Visualizing each layer is simple as we can specify aesthetic mapping and don't need to care about how data was converted to the actual visual values. Different layers can be progressively added to the plot to integrate different visualization methods for different data sets and thus leading to a personalized and complex tree graphic. Here, as an example, we reproduce Fig.2 of (Morgan, Segata, and

Huttenhower 2013) to demonstrate how to combine different data graphic layers to a circular phylogenetic tree using *ggtreeExtra*. The data sets[7] contains the relative abundance of bacteria (continuous data) at different body sites (categorical data). The associated data sets were imported by the *data* parameter of the *geom_fruit* function and displayed by corresponding geometric functions.

The tree was first visualized and annotated by *ggtree* with symbolic points to differentiate commensal microbes and potential pathogens (Fig. S7A), grey background to highlight different clades, and the clades were labeled by corresponding names (Fig. S7B). Then a heatmap was used to display the abundance of the microbes at different body sites. Heatmap cells are filled with different colors to represent different sampling sites and the transparency of the heatmap cells indicates the corresponding abundance of the microbes (Fig. S7C). Finally, a stacked bar chart was employed to display the relative abundance of the most abundant species at different body sites (Fig. S7D). The color of the bar chart represents different body sites and it is consistent with the color used in the heatmap. Fig. S7A-D were produced by adding multiple layers on the tree with a step-by-step solution. Source code for each layer is relatively simple and easy to follow. The combination of these layers eventually creates a compact graphic that represents complex information from several data sets. The *ggtreeExtra* package works seamlessly with *ggtree*, and a tree annotated by *ggtree* (Fig. S7A-B) can be used in *ggtreeExtra* to add data graphic layers on external panels and vice versa.

```r
library(ggtreeExtra)
library(ggtree)
library(treeio)
library(tidytree)
library(ggstar)
library(ggplot2)
library(ggnewscale)
library(patchwork)
tree <- read.tree("../data/HMP_tree/hmptree.nwk")
# the abundance and types of microbes
dat1 <- read.csv("../data/HMP_tree/tippoint_attr.csv")
# the abundance of microbes at different body sites.
dat2 <- read.csv("../data/HMP_tree/ringheatmap_attr.csv")
# the abundance of microbes at the body sites of greatest prevalence.
dat3 <- read.csv("../data/HMP_tree/barplot_attr.csv")
# adjust the order,it is optional.
dat2$Sites <- factor(dat2$Sites, levels=c("Stool (prevalence)", "Cheek (prevalence)",
                                          "Plaque (prevalence)","Tongue (prevalence)",
                                          "Nose (prevalence)", "Vagina (prevalence)",
                                          "Skin (prevalence)"))
dat3$Sites <- factor(dat3$Sites, levels=c("Stool (prevalence)", "Cheek (prevalence)",
                                          "Plaque (prevalence)", "Tongue (prevalence)",
                                          "Nose (prevalence)", "Vagina (prevalence)",
                                          "Skin (prevalence)"))
# extract the clade label information. Because some nodes of tree are annotated to genera,
# which can be displayed with high light using ggtree.
# This is optional, since the node information are always not present.
nodeids <- nodeid(tree, tree$node.label[nchar(tree$node.label)>4])
nodedf <- data.frame(node=nodeids)
nodelab <- gsub("[\\.0-9]", "", tree$node.label[nchar(tree$node.label)>4])
# The layers of clade and hightlight (optional)
poslist <- c(1.6, 1.4, 1.6, 0.8, 0.1, 0.25, 1.6, 1.6, 1.2, 0.4,
             1.2, 1.8, 0.3, 0.8, 0.4, 0.3, 0.4, 0.4, 0.4, 0.6,
             0.3, 0.4, 0.3)
labdf <- data.frame(node=nodeids, label=nodelab, pos=poslist)
# The circular layout tree.
p <- ggtree(tree, layout="fan", size=0.15, open.angle=5)
# add tip points with geom_star of ggstar
p <- p %<+% dat1 +
     geom_star(
         mapping=aes(fill=Phylum, starshape=Type, size=Size),
         starstroke=0.05
     ) +
```

[7] extracted from phyloxml file downloaded from https://github.com/biobakery/graphlan/tree/master/examples

```
    scale_fill_manual(
        values=c("#FFC125","#87CEFA","#7B68EE","#808080","#800080",
                 "#9ACD32","#D15FEE","#FFC0CB","#EE6A50","#8DEEEE",
                 "#006400","#800000","#B0171F","#191970"),
        guide=guide_legend(keywidth = 0.5, keyheight = 0.5, order=1,
        override.aes=list(starshape=15)),
        na.translate=FALSE) +
    scale_starshape_manual(
        values=c(15, 1),
        guide=guide_legend(keywidth = 0.5, keyheight = 0.5, order=2),
        na.translate=FALSE
    ) +
    scale_size_continuous(
        range = c(0.5, 1.5),
        guide = guide_legend(keywidth = 0.5, keyheight = 0.5, order=3,
        override.aes=list(starshape=15))
    ) +
    new_scale_fill() +
    theme(legend.position="none")
# optional for high light and clade labels
p1 <- p +
      geom_hilight(data=nodedf, mapping=aes(node=node),
                   extendto=6.8, alpha=0.3, fill="grey",
                   color="grey50", size=0.05
      ) +
      geom_cladelab(data=labdf,
                    mapping=aes(node=node, label=label, offset.text=pos),
                    barsize=NA, fontsize=0.7, angle="auto",
                    hjust=0.5, horizontal=FALSE, fontface="italic"
      )
# Heatmap, using geom_fruit to link geom_tile
p2 <- p1 +
      geom_fruit(
          data=dat2,
          geom=geom_tile,
          mapping=aes(y=ID, x=Sites, alpha=Abundance, fill=Sites),
          color = "grey50",
          offset = 0.04,
          size = 0.02
      )+
      scale_alpha_continuous(
          range=c(0, 1),
          guide=guide_legend(keywidth = 0.3, keyheight = 0.3, order=5)
      ) +
      scale_fill_manual(
          values=c("#0000FF","#FFA500","#FF0000","#800000",
                   "#006400","#800080","#696969"),
          guide=guide_legend(keywidth = 0.3, keyheight = 0.3, order=4)
      ) + theme(legend.position="none")
# Bar chart, using geom_fruit to link geom_col
p3 <- p2 +
      geom_fruit(
          data=dat3,
          geom=geom_col,
          mapping=aes(y=ID, x=HigherAbundance, fill=Sites),
          pwidth=0.38,
          orientation="y",
          position=position_stackx(),
      )+
      geom_treescale(fontsize=1.2, linesize=0.3, x=4.9, y=0.1) +
```

```
      theme(legend.position="none")
p4 <- (p + p1 + plot_layout(width=c(3.4,4)))/ (p2 + p3 + plot_layout(width=c(3.4,4))) +
      plot_layout(heights=c(3,4)) + plot_annotation(tag_levels = 'A')
p4
```

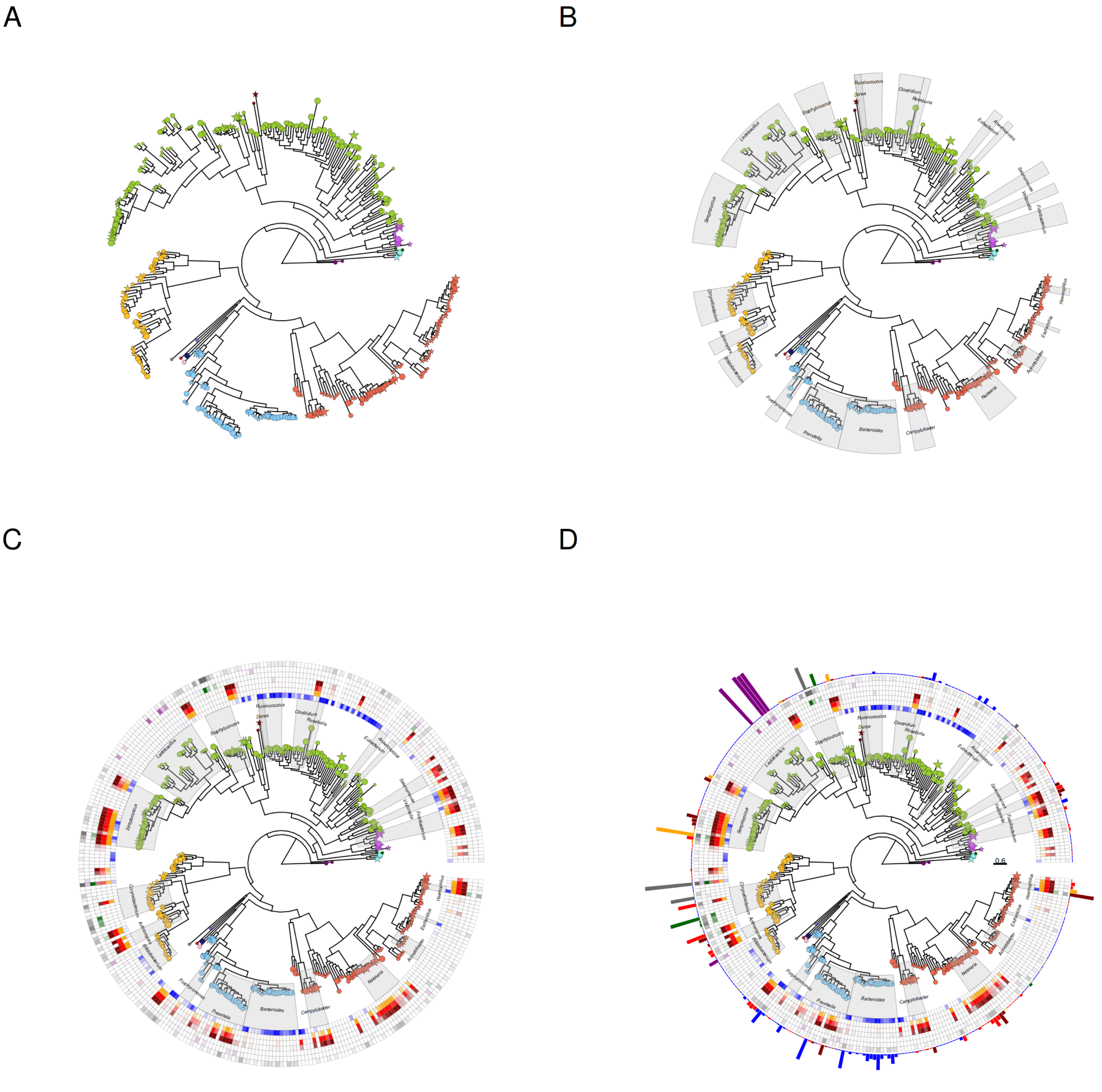

Fig. S7: **The abundance of microbes at different sites of human**. The shapes of symbolic points on the tips indicate the commensal microbes or potential pathogens (A). Clades were highlighted with grey background and labeled by clade name using *ggtree* (B). A heatmap layer represents different body sites the transparency of the colors indicates the abundance of microbes (C). The bar chart visualizes the relative abundance of the most abundant species at different body sites (D). Different graphic layers were added to the tree progressively to create the final figure (D).

4.3 Example of complex tree annotations

The *ggtreeExtra* package enables us to link *ggtree* (Yu et al. 2017) and geometric layers defined in the *ggplot2* community (Tab. S2 and Fig. S1). Different annotation layers to visualize high-dimensional associated data sets can be combined freely to create a complex tree graphic. As demonstrated in Fig S7, visualizing a data graphic layer is simple and multiple layers can be added progressively in *ggtreeExtra*.

Here, we reproduce Fig 2 of (Asnicar et al. 2015). The phylogenetic tree was built using a part of 3737 microbes (Segata et al. 2013). The associated data sets were extracted from GraPhlAn configuration file. They contain information of status (present or absent) and types of ATP synthesis genes, the type and capability of fatty acid metabolism, and the lengths of microbes genomes. A heatmap layer was added to display the presence statuses (discrete variable) of the two types (F-type and V/A-type) of ATPase. Another heatmap was added to visualize different types (as different colors) and capability (as transparency of the heatmap) of fatty acid metabolism. Finally, a bar chart colored by phylum information was employed to represent the lengths of the genomes. These layers were added progressively to the phylogenetic tree decorated with different symbolic points to indicate phylum information of the microbes, and background colors and labels for different clades (Fig. S8).

Notably, Proteobacteria and Spirochaetes were set to identical color in the original configuration file (Asnicar et al. 2015). Since the phylum information of all the tips was replaced by color code, we cannot separate the tips from Proteobacteria and Spirochaetes. This is a drawback of the GraPhlAn configuration file as it mixes associated data with the graphic setting. Some of the information was replaced by visual values and it is tedious, error-prone, and sometimes impossible to convert back to original data.

```r
library(ggtreeExtra)
library(ggtree)
library(treeio)
library(tidytree)
library(ggstar)
library(ggplot2)
library(ggnewscale)

tree <- read.tree("../data/kegg/kegg.nwk")
# The attributes of tip point
dt1 <- read.csv("../data/kegg/tippoint_attr.csv")
# The attributes of first ring
dt2 <- read.csv("../data/kegg/firstring_attr.csv")
# The attributes of second ring
dt3 <- read.csv("../data/kegg/secondring_attr.csv")
# The attrihutes of bar plot
dt4 <- read.csv("../data/kegg/barplot_attr.csv")

# To combine Spirochaetes and Proteobacteria, Since they have same color
# in original file, which cause some tips confused.
dt1 <- aggregate(.~ID, dt1, paste0, collapse="/")
#reorder the Phyla column
dt1$Phyla <- factor(dt1$Phyla, levels=c("Actinobacteria","Aquificae","Bacteroidetes",
                                        "Chlamydiae","Chlorobi","Chloroflexi","Crenarchaeota",
                                        "Cyanobacteria","Euryarchaeota","Firmicutes",
                                        "Spirochaetes/Proteobacteria",
                                        "Tenericutes","Thermi","Thermotogae","Other"))

# reorder the Type2 column
dt3$Type2 <- factor(dt3$Type2, levels=c("FA synth init", "FA synth elong",
                                        "acyl-CoA synth", "beta-Oxidation",
                                        "Ketone biosynth"))

# extract node label for the clade layers
nodelab <- tree$node.label[nchar(tree$node.label)>0]
nodeids <- nodeid(tree, nodelab)
# the position of clade label
textex <- c(1.0, 0.4, 0.2, 1.4, 1.4, 0.4, 1.4, 1.4, 0.4, 0.4,
            0.8, 1, 0.6, 0.6, 0.4, 0.3, 0, 0.4, 0.1, 0.25,
```

```
             0.2, 0.3, 0.8, 0.8, 0.8, 0.6, 2.4)
# optional for clade layers
cladelabels <- mapply(function(x, y, z){geom_cladelabel(node=x, label=y, barsize=NA, extend=0.3,
                                             offset.text=z, fontsize=1.3, angle="auto",
                                             hjust=0.5, horizontal=FALSE, fontface="italic")},
                                    nodeids, nodelab, textex, SIMPLIFY=FALSE)

# optional high light layers
fills <- c("#808080", "#808080", "#808080", "#808080", "#808080",
           "#191970", "#87CEFA", "#FFC125", "#B0171F", "#B0171F",
           "#B0171F", "#B0171F", "#B0171F", "#B0171F", "#B0171F",
           "#B0171F", "#B0171F", "#B0171F", "#B0171F", "#B0171F",
           "#B0171F", "#B0171F", "#9ACD32", "#9ACD32", "#9ACD32",
           "#006400", "#800000")
# optional for hight light
highlights <- mapply(function(x, y){geom_hilight(node=x, extendto=5.8, alpha=0.3,
                                                fill=y, color=y, size=0.05)},
                     nodeids, fills, SIMPLIFY=FALSE)

# to reproduce the original figures, we use the same colors.
# uses can custom set it.
colors <- c("#9ACD32", "#EE6A50", "#87CEFA", "#FFC125", "#D15FEE", "#8DEEEE", "#800000",
            "#006400", "#800080", "#808080", "#B0171F", "#191970", "#7B68EE",
            "#00CD00", "Black")
p1 <- ggtree(
          tree,
          layout="circular",
          size=0.1
      )
# Optional for hight clade
p1 <- p1 +
      highlights

p2 <- p1 %<+% dt1 +
     geom_tippoint(
         mapping=aes(
                     fill=Phyla
                 ),
         shape=21,
         size=1.2,
         stroke=0.05,
         position="identity",
         show.legend=FALSE
    )+
    scale_fill_manual(values=colors)
# Optional for clade label
p2 <- p2 +
    cladelabels +
    new_scale_fill()

p3 <- p2 +
      geom_fruit(
        data=dt2,
        geom=geom_tile,
        mapping=aes(
                    y=ID,
                    x=ring,
                    fill=Type1
                ),
        offset=0.01,
```

```
        pwidth=0.14
    ) +
    scale_fill_manual(
        name="ATP synthesis",
        values=c("#339933", "#dfac03"),
        guide=guide_legend(keywidth=0.35, keyheight=0.35, order=1)
    ) +
    new_scale_fill()

p4 <- p3 +
      geom_fruit(
          data=dt3,
          geom=geom_tile,
          mapping=aes(
                      y=ID,
                      alpha=Abundance,
                      x=Type2,
                      fill=Type2
                ),
          offset=0.001,
          pwidth=0.18
      ) +
      scale_fill_manual(
          name="Fatty Acid metabolism",
          values=c("#b22222", "#005500", "#0000be", "#9f1f9f", "#793a07"),
          guide=guide_legend(keywidth=0.35, keyheight=0.35, order=2)
      ) +
      scale_alpha_continuous(
          range=c(0, 0.4),
          guide=guide_legend(keywidth=0.35, keyheight=0.35,order=3)
      ) +
      new_scale_fill()

p5 <- p4 +
      geom_fruit(data=dt4,
           geom=geom_bar,
           mapping=aes(
                        y=ID,
                        x=Length,
                        fill=Phyla
                       ),
           stat="identity",
           orientation="y",
           pwidth=0.3,
           position=position_dodgex()) +
      scale_fill_manual(
           values=colors,
           guide=guide_legend(keywidth=0.35, keyheight=0.35, order=4)
      ) +
      geom_treescale(fontsize=1.2, linesize=0.3) +
      theme(legend.position=c(0.93, 0.76),
            legend.background=element_rect(fill=NA),
            legend.title=element_text(size=6),
            legend.text=element_text(size=4.5),
            legend.spacing.y = unit(0.02, "cm"))
p5
```

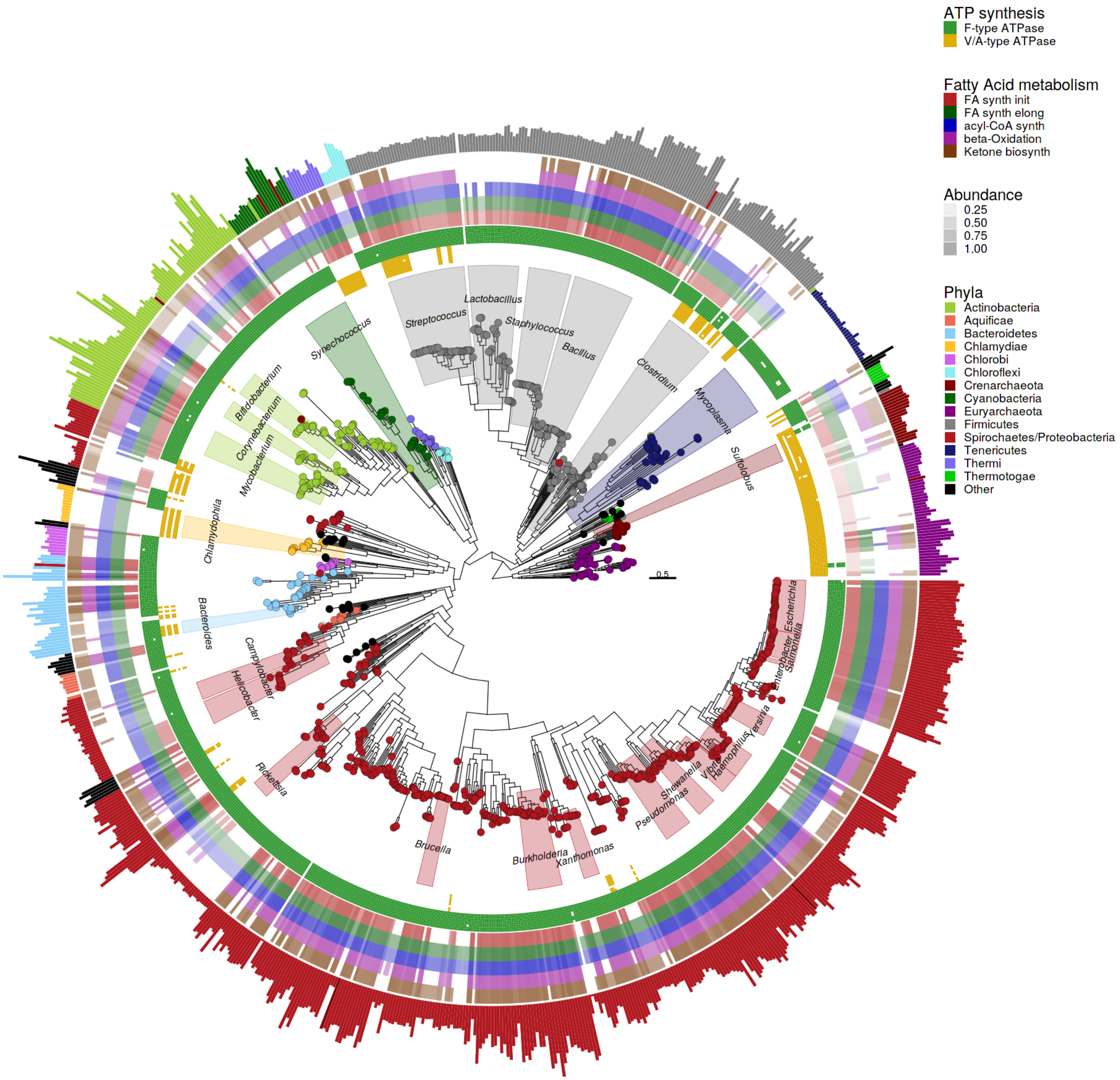

Fig. S8: **The phylogenetic tree was built using 963 microbial genomes (a part of 3737 microbes (Segata et al. 2013))**. A heatmap layer to represent the capability of ATP synthesis (two rings for two types of ATPase), another heatmap layer represents the capability of fatty acid metabolism (five rings for five types of genes (different colors, discrete variable) and their abundance (different transparency level, continuous variable)) and a bar chart layer represents the lengths of the genomes colored by phylum information.

4.4 Complex tree annotations combined with chord diagram

The Fig. 1b and Fig. 2 of (Helfrich et al. 2018) show the directional interactions and the biosynthetic potential of isolates from the *Arabidopsis* leaf microbiome on the phylogenetic tree using GraPhlAn (Asnicar et al. 2015). Since GraPhlAn only supports annotation on circular layout trees using a few geometric layers, some phylogenetic patterns behind multi-dimensional data can not be found easily. Here, we use *ggtree* and *ggtreeExtra* to combine and visualize the information presented in the Fig.1b and Fig.2 of (Helfrich et al. 2018) to a single tree graphic (Fig. S9). In detail, the tree was visualized using *inward_circular* layout and the interactions of the isolates were visualized as a chord diagram using *geom_taxalink* implemented in *ggtree* (Yu et al. 2017). The biosynthetic potential of the isolates was displayed as a heatmap. The types of the BGCs (biosynthesis gene

clusters) were used to color the heatmap and the numbers of BGCs were used to set the transparency of the heatmap. The number of interactions of inhibitions or sensitivities per strain was displayed by a stacked bar chart which was colored by the type of the interactions.

Chord diagram allows us to display flows or connections between taxa, such as syntenic linkage among genes and genomes, and reticulate evolutionary relationships including horizontal gene transfer. With the modular design (Fig. S1), *ggtreeExtra* allows more data types from diverse sources to be integrated with a phylogenetic tree and a chord diagram to explore associated data at the evolutionary and interaction context.

```
library(ggtree)
library(ggtreeExtra)
library(ggplot2)
library(ggstar)
library(MicrobiotaProcess)
library(ggnewscale)
library(grid)

alltax <- read.csv("../data/Arabidopsis_leaf_microbiome/all_stain_taxonomy.csv")
linktab <- read.csv("../data/Arabidopsis_leaf_microbiome/Interaction_link_tab.csv")
weighttab <- read.csv("../data/Arabidopsis_leaf_microbiome/Interaction_weight.csv")
tippoint <- read.csv("../data/Arabidopsis_leaf_microbiome/stain_tippoint.csv")
BGCsda <- read.csv("../data/Arabidopsis_leaf_microbiome/BGCs_heatmap.csv")

tippoint$Taxa <- factor(tippoint$Taxa,
                        levels=c("Actinobacteria",
                                 "Bacteroidetes",
                                 "Firmicutes",
                                 "Deinococcus-Thermus",
                                 "Alphaproteobacteria",
                                 "Betaproteobacteria",
                                 "Gammaproteobacteria"
                               )
                        )
tippoint$names <- gsub("s__Leaf","",tippoint$Isolation)

BGCsda$BGCs <- factor(BGCsda$BGCs,
                     levels=c("modular.PKS",
                              "modular.PKS.NRPS.hybrid",
                              "non_modular.PKS", "NRPS",
                              "RiPP",
                              "Quorum.sensing",
                              "terpene",
                              "other"
                            )
                    )
BGCsda$Count <- log2(BGCsda$Count+1)

trda <- convert_to_treedata(alltax)
p <- ggtree(trda, layout="inward_circular", size=0.2, xlim=c(18,NA))
p <- p %<+% tippoint

p1 <- p +
      geom_tippoint(
          mapping=aes(
                      color=Taxa,
                      shape=Level
                  ),
          size=1,
          alpha=0.8
      ) +
      scale_color_manual(values=c("#EF3B2C", "#1D91C0", "#FEB24C", "grey60",
```

```
                                   "#7FBC41", "#4D9221", "#276419"),
                          guide=guide_legend(
                                    keywidth=0.5,
                                    keyheight=0.5,
                                    order=2,
                                    override.aes=list(shape=c("Actinobacteria"=20,
                                                              "Bacteroidetes" =20,
                                                              "Firmicutes"    =20,
                                                              "Deinococcus-Thermus" =20,
                                                              "Alphaproteobacteria" =18,
                                                              "Betaproteobacteria"  =18,
                                                              "Gammaproteobacteria" =18
                                                              ),
                                                      size=2
                                             ),
                                    na.translate=TRUE
                                )
      ) +
      scale_shape_manual(values=c("Phylum"=20, "Class"=18), guide="none" )

p2 <- p1 +
      new_scale_color() +
      geom_taxalink(
          data=linktab,
          mapping=aes(
                      taxa1=Inhibitor,
                      taxa2=Sensitive,
                      color=Interaction
                  ),
          alpha=0.6,
          offset=0.1,
          size=0.15,
          ncp=10,
          hratio=1,
          arrow=grid::arrow(length = unit(0.005, "npc"))
      ) +
      scale_colour_manual(values=c("chocolate2", "#3690C0", "#009E73"),
                           guide=guide_legend(
                                      keywidth=0.8, keyheight=0.5,
                                      order=1, override.aes=list(alpha=1, size=0.5)
                           )
      )

p3 <- p2 +
      geom_fruit(
          data=BGCsda,
          geom=geom_star,
          mapping=aes(
                      y=Strain,
                      x=BGCs,
                      size=Count,
                      fill=BGCs
                  ),
          starshape = 13,
          starstroke = 0,
          offset=-0.9,
          pwidth=1.4,
          grid.params=list(linetype=3)
      ) +
      scale_size_continuous(range=c(0, 2),
```

```r
                           limits=c(sort(BGCsda$Count)[2], max(BGCsda$Count)),
                           breaks=c(1, 2, 3),
                           name=bquote(paste(Log[2],"(",.("Count+1"), ")")),
                           guide=guide_legend(keywidth = 0.4, keyheight = 0.4, order=4,
                                              override.aes = list(starstroke=0.3))
      ) +
      scale_fill_manual(
          values=c("#66C2A5", "#FC8D62", "#8DA0CB", "#E78AC3",
                   "#A6D854", "#FFD92F", "#E5C494", "#B3B3B3"),
          guide=guide_legend(keywidth = 0.4, keyheight = 0.4, order=3)
      )

p4 <- p3 +
      geom_tiplab(
          mapping=aes(
                      label=names
                  ),
          align=TRUE,
          size=1,
          linetype=NA,
          offset=9.3
      )

p5 <- p4 +
      new_scale_fill() +
      geom_fruit(
          data=weighttab,
          geom=geom_bar,
          mapping=aes(
                      x=value,
                      y=Strain,
                      fill=Number
                  ),
          stat="identity",
          orientation="y",
          offset=0.48,
          pwidth=2,
          axis.params=list(
                           axis = "x",
                           text.angle = -45,
                           hjust = 0,
                           vjust = 0.5,
                           nbreak = 4
                      )
      ) +
      scale_fill_manual(
          name = "Number of interactions",
          values=c("#E41A1C", "#377EB8", "#4DAF4A", "#984EA3"),
          guide=guide_legend(keywidth=0.5,keyheight=0.5,order=5)
      ) +
      theme(
            legend.background=element_rect(fill=NA),
            legend.title=element_text(size=6.5),
            legend.text=element_text(size=5),
            legend.spacing.y = unit(0.02, "cm"),
            legend.margin=margin(0.1, 0.9, 0.1,-0.9, unit="cm"),
            legend.box.margin=margin(0.1, 0.9, 0.1, -0.9, unit="cm"),
            plot.margin = unit(c(-1.2, -1.2, -1.2, 0.1),"cm")
      )
p5
```

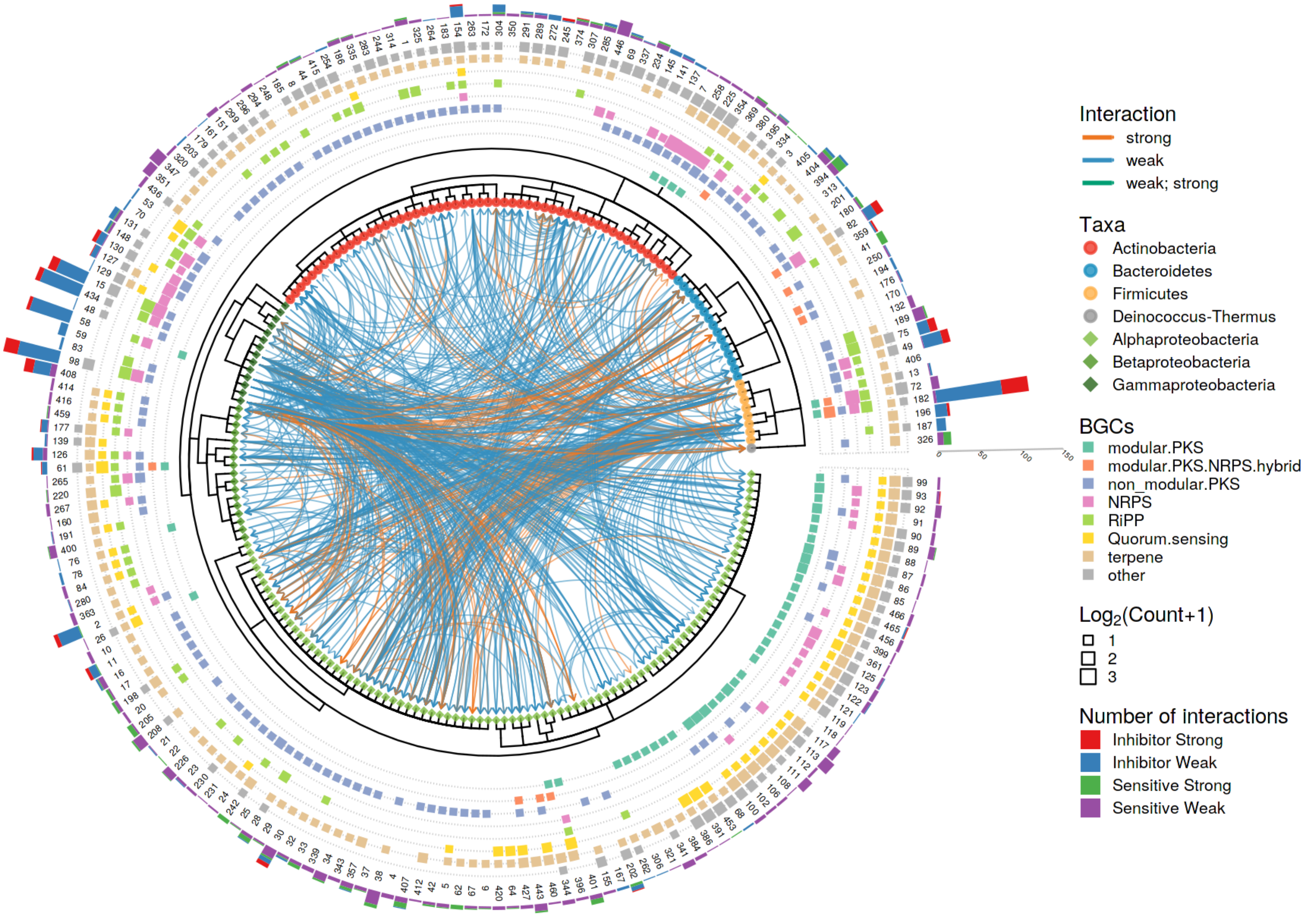

Fig. S9: **Illustration of representing multi-dimensional data sets on a inward circular phylogenetic tree with chord diagram incorporated to display inter-relationships.** The inward circular layout tree reflects the evolutionary relationships among isolates from the *Arabidopsis* leaf microbiome (Bai et al. 2015). In the center of the plot, the arcs connect isolates to show the inhibitory interactions, the arc arrows were meant to indicate directional inhibition and the colors of the arcs represent interaction strengths (*i.e.* weak or strong). The colors of symbolic points on tips indicate taxonomy annotation and circle points represent the phylum while square points represent the class from Proteobacteria. The heatmap (dot plot) on the external ring displays the number of detected BGCs (biosynthesis gene clusters). The outermost ring uses the stacked bar chart to visualize the number of inhibiting and sensitive interactions per strain.

With the visualization methods provided in ggtree and ggtreeExtra, it is more easy to identify evolutionary patterns. From Fig. S9, we found some strains from Firmicutes and Gammaproteobacteria have more inhibitor interactions. However, many strains from Alphaproteobacteria and Betateobacteria prefer the interaction of sensitivity. After subsequent Mann-Whitney U test, the number of different interactions among these strains was confirmed to be significant (highlighted in red in Fig. S10). To our knowledge, there are no other software tools that can easily produce a figure like this (Fig. S9) and the visualization do help us to explore the data and generate new insights as our findings were not revealed in the original paper (Helfrich et al. 2018).

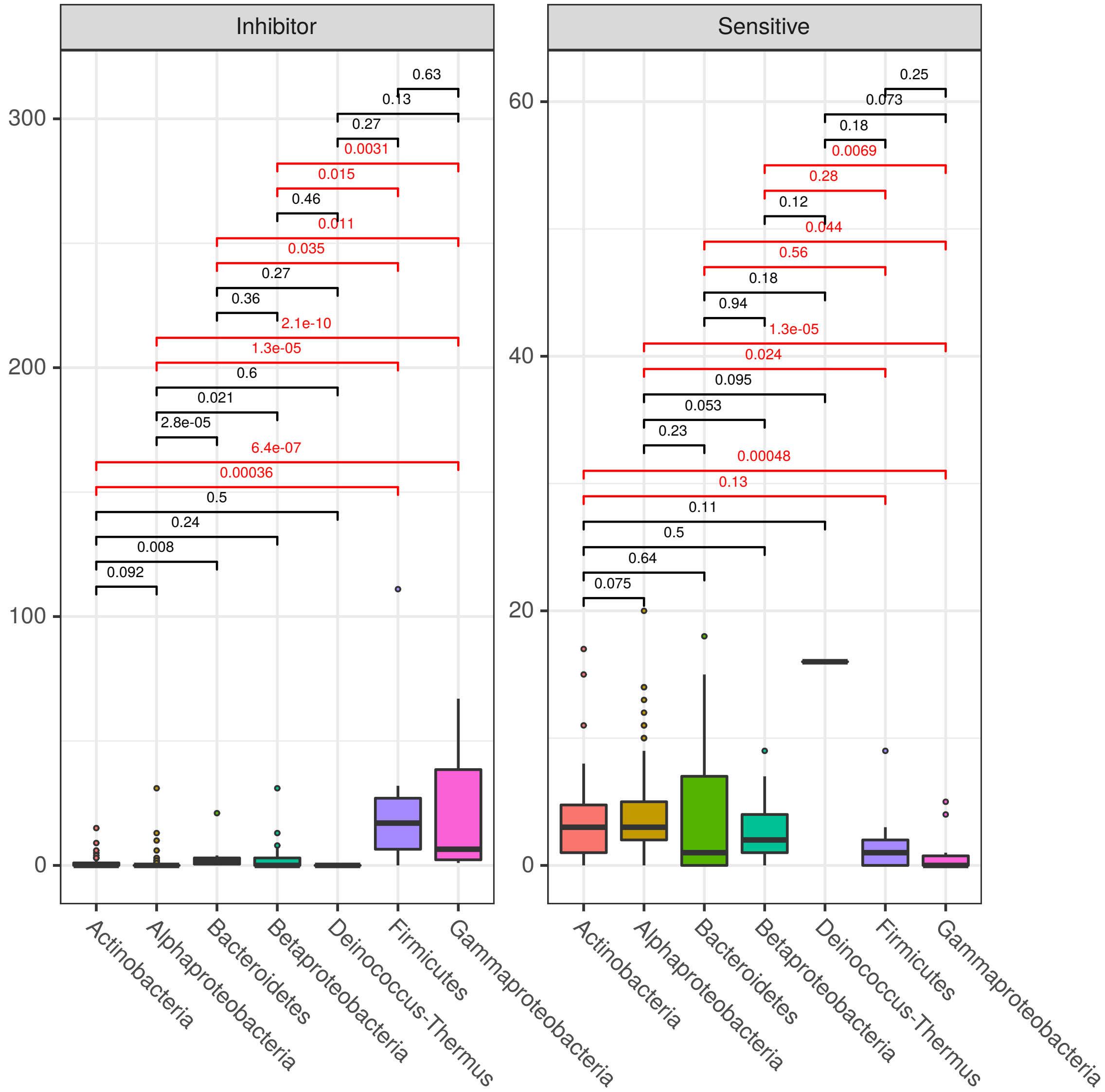

Fig. S10: **The comparison of number of inhibitor or sensitive interactions between the strains from different Phyla or Classes.**

5 Summary

The *ggtreeExtra* is a sub-package of the *ggtree* package suite and it takes all the advantages of *ggtree* (for tree visualization and annotation) and the *ggplot2* community (for data layers). Compare to other tools, *ggtreeExtra* supports more visualization methods implemented as graphic layers in different *ggplot2* extension packages and the combination of these layers creates more possibility to produce complex tree graphics. The *ggtreeExtra* has many features that cannot be found in other software tools, including:

1. Supports many visualization methods (*i.e.*, geometric layers developed by the *ggplot2* community) (Tab. S2)
2. Has no restriction of input data type and how the data was plotted (Tab. S2 and Session 4.1)
3. Supports more tree layouts compare to other tools and different layouts can be converted from each other (Fig. S3 and S4)
4. Able to annotate taxa with graphic information (Fig. S5)
5. Works with *treeio* package and allows evolutionary statistics inferred by commonly used software to be integrated and visualized with external data (Fig. S6)
6. Supports the grammar of graphics syntax (Fig. S7)
7. Able to combine different geometric layers (Fig. S8)
8. Supports tree with richly annotated layers generated by *ggtree* (*e.g.*, a tree with a chord diagram) (Fig. S9)

These features ensure that *ggtreeExtra* is more powerful to explore phylogenetic data and allows it to serve as a general tool that can be applied to different disciplines.

6 Methods

Code development. The *ggtreeExtra* package was designed with four parts: data importation, data scaling, external layer position adjustment and the API interface. External data integrated to the tree or *ggtree* objects (Yu et al. 2018; Yu 2020) can be directly used in *ggtreeExtra*. The *geom_fruit* function also allows directly importing data and the data will be reordered according to the tree structure. External data either integrated into the tree or imported by the geom_fruit function need to be rescaled, since the ranges of the data and the tree are not in the same order of magnitude. Before visualizing the data using a specified geometric layer, the data scaling will be performed automatically to standardize the data range according to the magnitude of the tree scale. The data scaling process only changes the range of the data while keeps the distribution of the data unchanged. This ensures the control of the panel width and avoids the tree and any external panel being squeezed (Fig. S1).

The position of the data graph (*i.e.* on the *right-hand* side or external ring of the tree, see also Fig. S3) is controlled by the *position* parameter, which accepts a *position* method. Follow the object-oriented framework of the *ggplot2* (Wickham 2016) package, *ggtreeExtra* implemented several *position* objects and methods including *position_identityx*, *positon_stackx*, *position_dodgex*, *position_dodgex2*, *position_points_jitterx*, *position_raincloudx* and *position_points_sinax* to shift the position of the corresponding geometric layer horizontally. To increase user experience, the default value of the *position* parameter is set to "auto" and the *geom_fruit* function will guess and determine a suitable position object for the specified geometric layer. That means using *positon_stackx* for *geom_bar* and *geom_col*, *positon_dodgex* for *geom_boxplot* and *geom_violin*, *geom_identityx* for others (*e.g. geom_point*, *geom_tile*, *geom_star*, *geom_phylopic etc.*). A geometric layer defined in *ggplot2*, *ggtree* or other *ggplot2*-extensions that has a *position* parameter should be compatible with the *geom_fruit* function, since it allows using *position* method defined in the *ggtreeExtra* package to adjust the output layer position (Tab. S2).

The interface of the geom_fruit is intuitive. It is easy to follow and working in the way users expect it to. External data can be imported via the *data* argument. The data will be visualized using a geometric layer, which can be specified via the *geom* argument. The *mapping* argument is designed to construct aesthetic mappings that describe how variables in the data are mapped to visual characteristics (*e.g.* color, shape, and size, *etc.*). The *y* aesthetic is required to be mapped to a variable that stored the taxa names in the external data set to allow geom_fruit to link through the data and the tree. The data will be reordered based on the tree structure. Other aesthetic mappings and non-variable settings can be specified to control the appearance of the data graph. Users can use the *offset* and *pwidth* arguments respectively to specify the distance between the external panels and the width of the data graph layer. Besides, the *geom_fruit* function allows setting axis and background grid lines for the data graph layer using the *grid.params* and *axis.params* arguments respectively. The data graph layer produced by *geom_fruit* will be displayed on the external panel and perfectly aligned to the tree. Visualizing data in *ggtreeExtra* is as simple as visualizing the data in *ggplot2* (Wickham 2016). The critical elements are the same – a data frame and a geometric layer to visualize the data with specific aesthetic mapping. Users don't need to care about how the tree was linked to the data, since the *geom_fruit* function will do all the data manipulation for us. The *geom_fruit* was developed using object-oriented programming and the output data layer can be added to the tree graph using the plus sign that followed the layered grammar of graphics. Different data sets can be progressively added and displayed on different external panels.

NOTE: source codes and datasets to produce this file can be obtained online[8].

References

Aphalo, Pedro J. 2020. *Ggpmisc: Miscellaneous Extensions to 'Ggplot2'.* https://CRAN.R-project.org/package=ggpmisc.

Asnicar, Francesco, George Weingart, Timothy L Tickle, Curtis Huttenhower, and Nicola Segata. 2015. "Compact Graphical Representation of Phylogenetic Data and Metadata with Graphlan." *PeerJ* 3: e1029. https://doi.org/10.7717/peerj.1029.

Bai, Yang, Daniel B Müller, Girish Srinivas, Ruben Garrido-Oter, Eva Potthoff, Matthias Rott, Nina Dombrowski, et al. 2015. "Functional Overlap of the Arabidopsis Leaf and Root Microbiota." *Nature* 528 (7582): 364–69. https://doi.org/10.1038/nature16192.

Berger, Simon A., Denis Krompass, and Alexandros Stamatakis. 2011. "Performance, Accuracy, and Web Server for Evolutionary Placement of Short Sequence Reads under Maximum Likelihood." *Systematic Biology* 60 (3): 291–302. https://doi.org/10.1093/sysbio/syr010.

Boussau, Bastien, Gergely J. Szöllősi, Laurent Duret, Manolo Gouy, Eric Tannier, and Vincent Daubin. 2013. "Genome-Scale Coestimation of Species and Gene Trees." *Genome Research* 23 (2): 323–30. https://doi.org/10.1101/gr.141978.112.

Drummond, Alexei J, and Andrew Rambaut. 2007. "BEAST: Bayesian Evolutionary Analysis by Sampling Trees." *BMC Evolutionary Biology* 7 (1): 1–8. https://doi.org/10.1186/1471-2148-7-214.

[8]https://github.com/YuLab-SMU/plotting-tree-with-data-using-ggtreeExtra

FC, Mike. 2020. *Ggpattern: Geoms with Patterns.*

Helfrich, Eric J. N., Christine M. Vogel, Reiko Ueoka, Martin Schäfer, Florian Ryffel, Daniel B. Müller, Silke Probst, Markus Kreuzer, Jörn Piel, and Julia A. Vorholt. 2018. "Bipartite Interactions, Antibiotic Production and Biosynthetic Potential of the Arabidopsis Leaf Microbiome." Journal Article. *Nature Microbiology* 3 (8): 909–19. https://doi.org/10.1038/s41564-018-0200-0.

Höhna, Sebastian, Michael J. Landis, Tracy A. Heath, Bastien Boussau, Nicolas Lartillot, Brian R. Moore, John P. Huelsenbeck, and Fredrik Ronquist. 2016. "RevBayes: Bayesian Phylogenetic Inference Using Graphical Models and an Interactive Model-Specification Language." *Systematic Biology* 65 (4): 726–36. https://doi.org/10.1093/sysbio/syw021.

Huerta-Cepas, Jaime, François Serra, and Peer Bork. 2016. "ETE 3: Reconstruction, Analysis, and Visualization of Phylogenomic Data." *Molecular Biology and Evolution* 33 (6): 1635–8. https://doi.org/10.1093/molbev/msw046.

Kumar, Sudhir, Glen Stecher, Michael Suleski, and S. Blair Hedges. 2017. "TimeTree: A Resource for Timelines, Timetrees, and Divergence Times." *Molecular Biology and Evolution* 34 (7): 1812–9. https://doi.org/10.1093/molbev/msx116.

Letunic, Ivica, and Peer Bork. 2019. "Interactive Tree of Life (iTOL) V4: Recent Updates and New Developments." *Nucleic Acids Research* 47 (W1): W256–W259. https://doi.org/10.1093/nar/gkz239.

Matsen, Frederick A, Robin B Kodner, and E Virginia Armbrust. 2010. "Pplacer: Linear Time Maximum-Likelihood and Bayesian Phylogenetic Placement of Sequences onto a Fixed Reference Tree." *BMC Bioinformatics* 11 (1): 538. https://doi.org/10.1186/1471-2105-11-538.

McMurdie, Paul J., and Susan Holmes. 2013. "Phyloseq: An R Package for Reproducible Interactive Analysis and Graphics of Microbiome Census Data." *PLoS ONE* 8 (4): e61217. https://doi.org/10.1371/journal.pone.0061217.

Mirarab, Siavash, and Tandy Warnow. 2015. "ASTRAL-II: Coalescent-Based Species Tree Estimation with Many Hundreds of Taxa and Thousands of Genes." *Bioinformatics (Oxford, England)* 31 (12): i44–52. https://doi.org/10.1093/bioinformatics/btv234.

Morgan, Xochitl C., Nicola Segata, and Curtis Huttenhower. 2013. "Biodiversity and Functional Genomics in the Human Microbiome." *Trends in Genetics* 29 (1): 51–58. https://doi.org/10.1016/j.tig.2012.09.005.

Pond, Sergei L. Kosakovsky, Simon D. W. Frost, and Spencer V. Muse. 2005. "HyPhy: Hypothesis Testing Using Phylogenies." *Bioinformatics* 21 (5): 676–79. https://doi.org/10.1093/bioinformatics/bti079.

Segata, Nicola, Daniela Börnigen, Xochitl C. Morgan, and Curtis Huttenhower. 2013. "PhyloPhlAn Is a New Method for Improved Phylogenetic and Taxonomic Placement of Microbes." Journal Article. *Nature Communications* 4 (1): 2304. https://doi.org/10.1038/ncomms3304.

Smith, Garrett J, and Kelly C Wrighton. 2019. "Metagenomic Approaches Unearth Methanotroph Phylogenetic and Metabolic Diversity." *Curr Issues Mol Biol* 33: 57–84. https://doi.org/10.21775/9781912530045.03.

Song, Se Jin, Jon G. Sanders, Frédéric Delsuc, Jessica Metcalf, Katherine Amato, Michael W. Taylor, Florent Mazel, et al. 2020. "Comparative Analyses of Vertebrate Gut Microbiomes Reveal Convergence Between Birds and Bats." Edited by Joerg Graf. *mBio* 11 (1). https://doi.org/10.1128/mBio.02901-19.

Stamatakis, Alexandros. 2014. "RAxML Version 8: A Tool for Phylogenetic Analysis and Post-Analysis of Large Phylogenies." *Bioinformatics*, January, btu033. https://doi.org/10.1093/bioinformatics/btu033.

Wang, Li-Gen, Tommy Tsan-Yuk Lam, Shuangbin Xu, Zehan Dai, Lang Zhou, Tingze Feng, Pingfan Guo, et al. 2020. "Treeio: An R Package for Phylogenetic Tree Input and Output with Richly Annotated and Associated Data." *Molecular Biology and Evolution* 37 (2): 599–603. https://doi.org/10.1093/molbev/msz240.

Wickham, Hadley. 2016. *Ggplot2: Elegant Graphics for Data Analysis.* Springer-Verlag New York. https://ggplot2.tidyverse.org.

Wilke, Claus O. 2020. *Ggridges: Ridgeline Plots in 'Ggplot2'.* https://CRAN.R-project.org/package=ggridges.

Wilkinson, Leland. 2012. "The Grammar of Graphics." In *Handbook of Computational Statistics: Concepts and Methods*, edited by James E. Gentle, Wolfgang Karl Härdle, and Yuichi Mori, 375–414. Berlin, Heidelberg: Springer Berlin Heidelberg. https://doi.org/10.1007/978-3-642-21551-3_13.

Yang, Ziheng. 2007. "PAML 4: Phylogenetic Analysis by Maximum Likelihood." *Molecular Biology and Evolution* 24 (8): 1586–91. https://doi.org/10.1093/molbev/msm088.

Yu, Guangchuang. 2020. "Using Ggtree to Visualize Data on Tree-Like Structures." *Current Protocols in Bioinformatics* 69 (1): e96. https://doi.org/10.1002/cpbi.96.

Yu, Guangchuang, Tommy Tsan-Yuk Lam, Huachen Zhu, and Yi Guan. 2018. "Two Methods for Mapping and Visualizing Associated Data on Phylogeny Using Ggtree." *Molecular Biology and Evolution* 35 (2): 3041–3. https://doi.org/10.1093/molbev/msy194.

Yu, Guangchuang, David Smith, Huachen Zhu, Yi Guan, and Tommy Tsan-Yuk Lam. 2017. "Ggtree: An R Package for Visualization and Annotation of Phylogenetic Trees with Their Covariates and Other Associated Data." *Methods in Ecology and Evolution* 8 (1): 28–36. https://doi.org/10.1111/2041-210X.12628.
